# Current status and perspectives of chimeric antigen receptor modified T cells for cancer treatment

**DOI:** 10.1007/s13238-017-0400-z

**Published:** 2017-05-02

**Authors:** Zhenguang Wang, Yelei Guo, Weidong Han

**Affiliations:** Molecular & Immunological Department, Bio-therapeutic Department, Chinese PLA General Hospital, Beijing, 100853 China

**Keywords:** chimeric antigen receptor, CAR-T, engineered T cells, adoptive cell therapy, cancer treatment

## Abstract

Chimeric antigen receptor (CAR) is a recombinant immunoreceptor combining an antibody-derived targeting fragment with signaling domains capable of activating cells, which endows T cells with the ability to recognize tumor-associated surface antigens independent of the expression of major histocompatibility complex (MHC) molecules. Recent early-phase clinical trials of CAR-modified T (CAR-T) cells for relapsed or refractory B cell malignancies have demonstrated promising results (that is, anti-CD19 CAR-T in B cell acute lymphoblastic leukemia (B-ALL)). Given this success, broadening the clinical experience of CAR-T cell therapy beyond hematological malignancies has been actively investigated. Here we discuss the basic design of CAR and review the clinical results from the studies of CAR-T cells in B cell leukemia and lymphoma, and several solid tumors. We additionally discuss the major challenges in the further development and strategies for increasing anti-tumor activity and safety, as well as for successful commercial translation.

## INTRODUCTION

“Natural forces within us are the true healers of disease.”—Hippocrates (de Coana et al., 2015).

Undoubtedly, the immune system is the right cancer healer, especially in the context of currently available therapies such as chemotherapy, radiotherapy, and targeted therapy, which have been less successful than anticipated. Harnessing the immune system to kill cancer is a durable concept that has more than 100 years of history; it was first demonstrated in 1891 by William Coley’s use of Coley’s toxin, a mixture of heat-killed bacteria to elicit regression of inoperable sarcomas (Elert, 2013). Despite this early beginning, efforts to reliably manipulate the immune system to promote tumor regression have been universally disappointing. In recent decades, with the significant progress in understanding the inherent immune biology related to cancer, effective immunotherapy treatments for cancer have gradually emerged (Fyfe et al., 1995; Atkins et al., 1999; Kantoff et al., 2010) and reached an important turnover in the history of cancer treatment as named by *Science* magazine the “breakthrough of 2013” due to the striking proof-of-concept data of immune checkpoint anti-CTLA-4 and PD-1 antibodies as well as CAR therapy (Couzin-Frankel, 2013). Subsequently, a spectrum of encouraging outcomes of those modalities in other tumors have attracted more big players during the past 2 years, denoting that cancer immunotherapy is coming of age.

**Figure 1:**
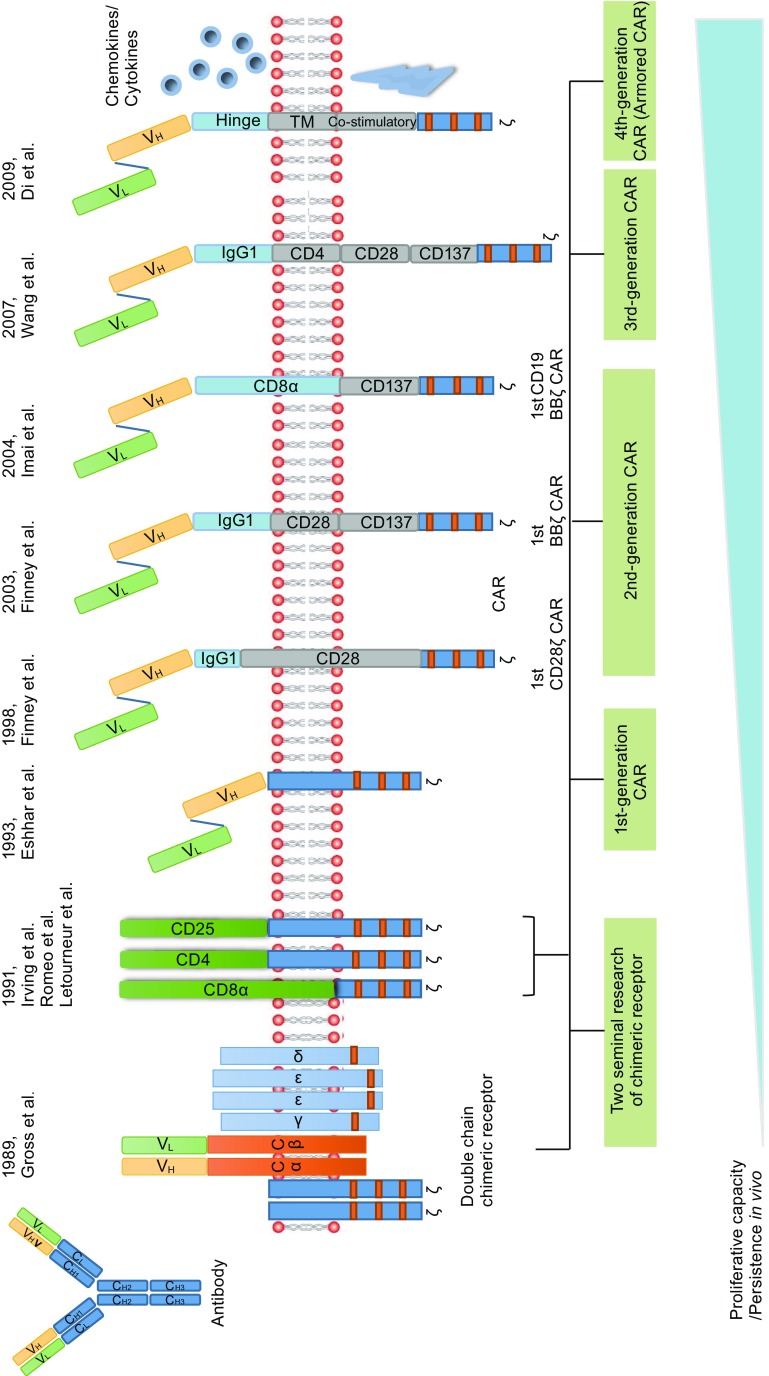
Evolution of CAR

The presented concept of CAR is based on two seminal research studies as the increasing understanding of the construct and function of T cell receptor (TCR) complex (Fig. 1). First, in 1989 Gross et al. constructed a chimeric TCR (cTCR) gene made by replacing the Vα and Vβ extracellular domains of the TCR chains with their V_H_ and V_L_ immunoglobulin homologs (CαV_H_ + CβV_L_ or CαV_L_ + CβV_H_). The resulting cTCR was expressed on the surface of cytotoxic T lymphocytes, recognized antigen in a non-MHC-restricted manner, and effectively transmitted the transmembrane signal for T cell activation (Gross et al., 1989). These results proved that replacing the variable region of TCR with those of antibody for endowing the T cells with antibody-type specificity is viable (Eshhar, 2014), and was subsequently followed by Goverman et al. with a consistent outcome (Goverman et al., 1990). Another pioneering study mainly focused on the chimeric proteins constructed between either CD8, CD4, or CD25 (also called α chain of the human interleukin-2 receptor) and cytoplasmic tails of ζ (Irving and Weiss, 1991; Romeo and Seed, 1991; Letourneur and Klausner, 1991). Those chimeric proteins have resulted in biochemical events of early T cell activation such as interleukin-2 (IL-2) production and Ca^2+^ influx, which validated that cytoplasmic tails of ζ could replicate much of the TCR signaling (van der Stegen et al., 2015). Taking advantage of these advances, in 1993 Eshhar et al. pioneered to design a gene composed of a single chain variable fragment (scFv) of an antibody linked with ζ chains, which is aimed to overcome the difficulty in activating anti-tumor T cells through the TCR (Eshhar et al., 1993). The transfected cytolytic T cell hybridoma triggered IL-2 secretion upon encountering antigen and mediated non-MHC-restricted hapten-specific target cell lysis. This new artificial receptor called T-body is known as the first-generation CAR. Subsequent experiments after this initial report further demonstrated the anti-tumor potential of the T cells transfected with these fusion receptors (Brocker et al., 1993; Hwu et al., 1993; Stancovski et al., 1993; Gross et al., 1995; Hwu et al., 1995). However, these fusion receptors are devoid of costimulatory elements that are required for full T cell activation and only induce limited cytokine production and cannot activate resting or naïve lymphocytes (Brocker and Karjalainen, 1995). Furthermore, in the absence of costimulatory signaling by CD28, resting T lymphocytes typically undergo anergy or apoptosis (Boussiotis et al., 1996). To address these issues, the introduction of costimulatory element CD28 (the best characterized costimulatory molecule) to the first-generation CAR was first described by Finney et al. in 1998. This second-generation CAR is capable of mediating up to 20 times more IL-2 production on stimulation with solid-phase Ag when compared to first-generation CAR. Moreover, constructs with the CD28 signaling domain proximal and the ζ -chain distal to the membrane were found to express more efficiently in Jurkat than constructs with the opposite orientation (Finney et al., 1998), thus determining the signaling element arranging pattern adopted by other researchers in the years since. Other than CD28, other costimulatory molecules such as CD134/CD137 also have been incorporated into the first-generation CAR by Finney et al. (2003). Second-generation CAR is superior for inducing cytokine production and proliferation of CAR-T cells compared to the first-generation CAR, which was proved in several preclinical studies (Haynes et al., 2002a, b; Imai et al., 2004; Kowolik et al., 2006) and was further verified in one clinical trial to directly compare such two generation CARs (Savoldo et al., 2011). The initial pilot clinical studies of CAR were opened in solid tumors (Lamers et al., 2006; Kershaw et al., 2006). However, substantial clinical efficacy has been shown in hematological malignancies treated with second-generation CARs (Kochenderfer et al., 2010; Porter et al., 2011; Kalos et al., 2011; Wang et al., 2014; Maude et al., 2014a; Davila et al., 2014; Dai et al., 2015; Lee et al., 2015a; Kochenderfer et al., 2015; Porter et al., 2015; Turtle et al., 2016a; Wang et al., 2016; Zhang et al., 2016a). Third-generation CAR contains two costimulatory domains, which result in more potent persistence and other T cell functions in preclinical studies (Wang et al., 2007; Zhong et al., 2010; Carpenito et al., 2009; Choi et al., 2014). However, the clinical benefit is not as good as expected (Till et al., 2012) and more studies are needed. To further improve the anti-tumor effect of CAR-T cells, engineering CAR-T cells to additionally express cytokines or co-stimulatory ligands (fourth-generation CAR, also called armored CAR) has been employed (Di Stasi et al., 2009) and actively researched (Pegram et al., 2014), yet no results of clinical trial have been published so far. In this article, we briefly review the common structure of CAR and emerging clinical activity, toxicities, and challenges of this novel technology.

## COMMON CAR STRUCTURES

**Figure 2:**
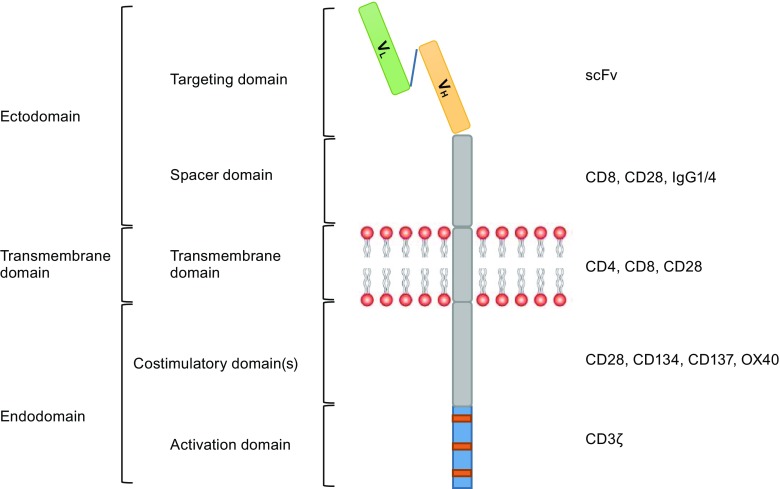
Anatomy of a second-generation CAR

CAR is artificial type I transmembrane protein assembled from a series of modular compositions including an amino terminal ectodomain and carboxy-terminal endodomain, as well as a transmembrane domain (TM) (Fig. 2) (Eshhar, 2008; Curran et al., 2012; Dai et al., 2016). Ectodomain usually consists of a target-binding domain most commonly derived from the scFv of a monoclonal antibody (mAb) specific for a surface molecule on the tumor cell (Eshhar et al., 1993; Kershaw et al., 2005), and a spacer (also known as a hinge) domain typically comprises immunoglobulin-like C_H2_-C_H3_ (Fc) domains from the constant region of immunoglobulin G (IgG) (Finney et al., 1998; Till et al., 2012), CD8 (Porter et al., 2011; Wang et al., 2014; Zhang et al., 2016a) or CD28 (Kochenderfer et al., 2010; Kochenderfer et al., 2015), which extends the antigen-binding domain out from the T cell membrane. Endodomain acts to transmit T cell signals and typically comprises 0 or 1 or 2 costimulatory domains such as CD28, CD134 (OX40) or CD137 (4-1BB) (van der Stegen et al., 2015; Finney et al., 1998, 2003) and activation domain representing the CD3 ζ (Ghorashian et al., 2015; Sadelain et al., 2013). Such synthetic tumor-targeting receptors provide a choice of specificity and controlled T cell activation that is mainly attributed to extracellular antigen-binding component and intracellular-signaling components that have received the most attention and have been well described (van der Stegen et al., 2015; Ghorashian et al., 2015; Sadelain et al., 2013; Gill and June 2015; Jackson et al., 2016). However, the spacer domain should not be overlooked; it is equally crucial for effective initiation of T cell signaling as it provides flexibility and optimizes T cell and target cell engagement by overcoming the structural constraints in T cells: target cell interactions (Guest et al., 2005; Hudecek et al., 2013, 2015; Srivastava and Riddell 2015). The optimal length of the spacer domain for each CAR may differ depending on the dimensions of the cell surface antigen that is targeted by the scFv (Harris and Kranz, 2016). The transmembrane domain is considered to be a purely structural requirement for anchoring the CAR to the cell membrane and has little to no effect on the function of CAR (van der Stegen et al., 2015).

By arming the T cells with CAR, the engineered T cells can directly recognize cancer cell surface antigens in an MHC-independent fashion and undergo activation, providing an alternative to conventional TCR and enabling them to circumvent the major hurdles suffered by cancer patients, including tumor escapes resulting from downregulation or loss of HLA expression as well as T cell anergy due to decreasing or loss of the expression of costimulatory molecules required for triggering the full potency of T cells (Gross and Eshhar, 2016). Compared to native TCR, scFv-based antigen recognition has both benefits and limitations. CAR only recognizes target antigens expressed on the cell surface rather than internal antigens that are processed and presented by the cells’ MHC, but various cell-surface molecules such as proteins, carbohydrate (Lewis-Y, TAG-72) (Peinert et al., 2010; Ritchie et al., 2013; Hombach et al., 1997; McGuinness et al., 1999), and glycolipid (GD2, GD3) structures (Pule et al., 2008; Louis et al., 2011; Rossig et al., 2001; Yun et al., 2000) can be recognized by CAR, which is a compensation for the limited target selection.

**Figure 3:**
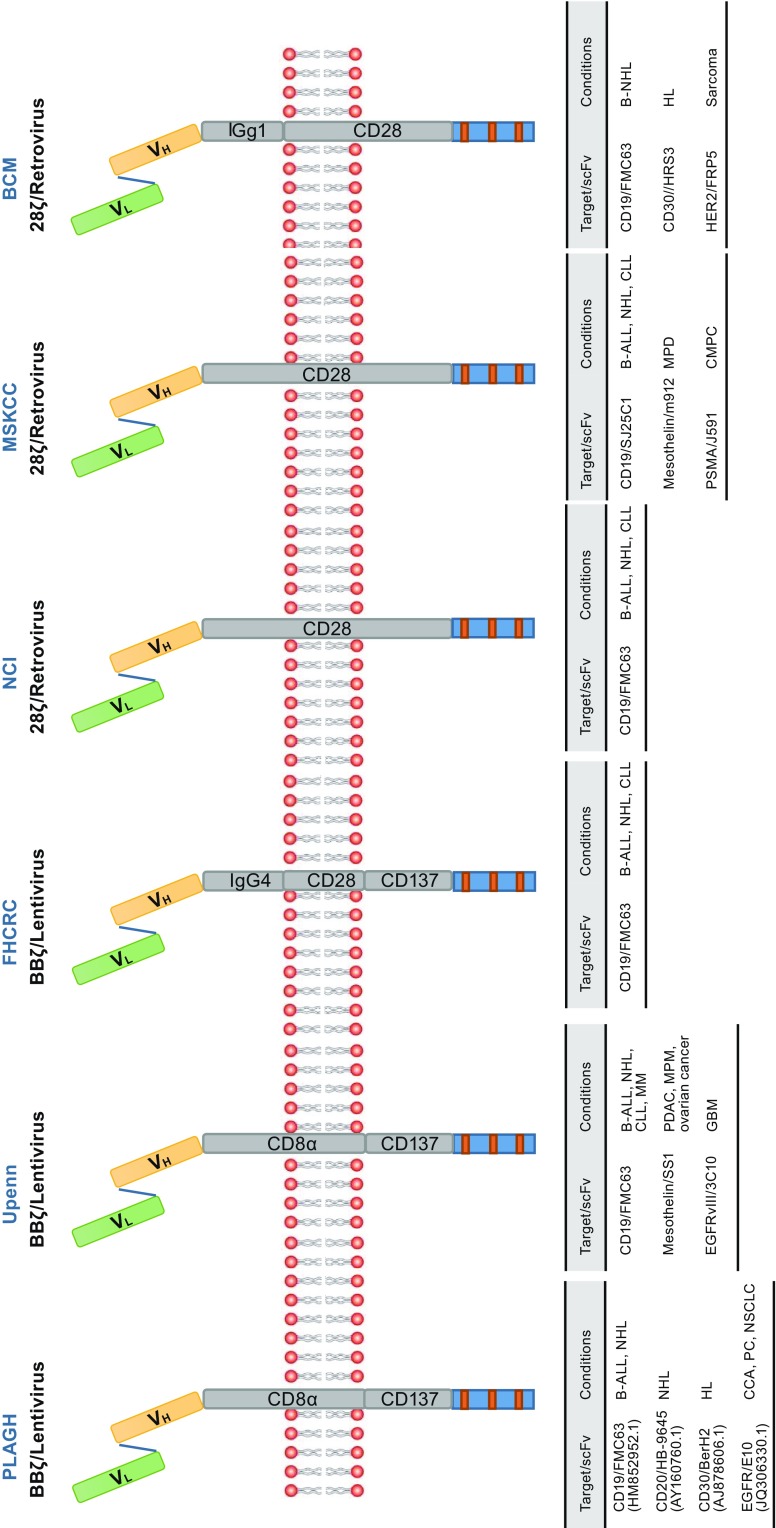
Common second-generation CARs. Abbreviations: B-ALL, B cell acute lymphoblastic leukemia; BCM, Baylor College of Medicine; B-NHL, B cell non -Hodgkin’s lymphoma; CCA, cholangiocarcinoma; CLL, chronic lymphocytic leukemia; CMPC, castrate metastatic prostate cancer; EGFR, epidermal growth factor receptor; EGFRvIII, variant III of the epidermal growth factor receptor; FHCRC, Fred Hutchinson Cancer Research Center; GBM, glioblastoma multiforme; HER2, human epidermal growth factor receptor-2; HL, Hodgkin’s lymphoma; MM, multiple myeloma; MPD, malignant pleural disease; MPM, malignant pleural mesothelioma; MSKCC, Memorial Sloan Kettering Cancer Center; NCI, National Cancer Institute; NSCLC, non-small cell lung cancer; PC, pancreatic cancer; PDAC, pancreatic ductal adenocarcinoma; PLAGH, Chinese PLA General Hospital; PSMA, prostate-specific membrane antigen; scFv, single chain variable fragment; Upenn, University of Pennsylvania

The CAR format provides an opportunity to recognize practically any desired target antigen by changing only the corresponding binding moiety while retaining the backbone structure. Moreover, owing to the modular design, sophisticated engineering of diverse domain components becomes possible. These unique features promote the versatility of CAR structures (Fig. 3); for instance, several second-generation CD19-specific CARs are being tested in clinical trials. The major structural difference between currently applying second-generation CARs is costimulatory domain (van der Stegen et al., 2015). CD28 costimulatory component has been used by the National Cancer Institute (NCI; USA) (Lee et al., 2015a), Memorial Sloan Kettering Cancer Center (MSKCC; USA) (Davila et al., 2014), and Baylor College of Medicine (BCM; USA) (Savoldo et al., 2011), while the 4-1BB costimulatory component has been incorporated by the University of Pennsylvania (Upenn; USA) (Maude et al., 2014a), Chinese PLA General Hospital (PLAGH; CHINA) (Wang et al., 2014; Dai et al., 2015; Zhang et al., 2016a), and the Fred Hutchinson Cancer Research Center (FHCRC; USA) (Turtle et al., 2016a). Accordingly, the second-generation CARs could be classified as two categories of receptors based on the costimulatory domain, referred to as 28ζ and BBζ CAR. Both 28ζ and BBζ CAR have been used to successfully treat multiple blood cancers (Zhang et al., 2015). BBζ CAR appears to favor persistence and memory T cell formation, while 28ζ CAR presents more potent cytotoxic activity and early tumor eradication. So, combining the benefits of 4-1BB and CD28 costimulation could be a good option to best optimize CAR (Holohan et al., 2015). Rather than the strategy of combining CD28 and 4-1BB costimulation, Zhao et al. demonstrated that 28ζ CAR-T cells that constitutively express 4-1BB ligand (4-1BBL) promote T cell expansion and tumor eradication while reducing exhaustion (Zhao et al., 2015), providing valuable implications for evolving CAR-T cell therapies. More studies are required to better understand the kinetics of each of the costimulatory domains and their relative clinical effects.

Besides the costimulatory component, variability is present in scFv fragments, hinge, and transmembrane domains, in addition to differences in CAR transduction approaches, so it can be difficult to compare results from different studies.

## CLINICAL OUTCOMES OF HEMATOLOGICAL MALIGNANCIES

Following a decade of preclinical optimization, CAR-T cell therapy has produced impressive clinical results in treating patients with relapsed or refractory B cell leukemia and lymphoma whose treatment options are limited and prognosis is poor. To date, there are more than 30 publications and a large number of congress abstracts reporting clinical trials of CAR-T cells in hematologic malignancies. Although the initial clinical evaluation of CAR-T cells focused on B cell non-Hodgkin’s lymphoma (B-NHL) (Kochenderfer et al., 2010; Till et al., 2008), the most striking outcomes have been obtained in B-ALL by targeting CD19, a B cell-lineage antigen expressed on the surface of normal B cells and many malignant B cells (Scheuermann and Racila, 1995; Depoil et al., 2008). Another pan-B cell marker, CD20, is also an attractive target for CAR-T cell therapy in B cell malignancies (Raufi et al., 2013). Preliminary clinical trials evaluating anti-CD20 CAR-T cells for patients with B-NHL revealed minimal toxicities with modest efficacy (Wang et al., 2014; Till et al., 2008, 2012), until recently a phase IIa clinical trial performed at PLAGH demonstrated an objective remission rate (ORR) of 82% (complete remission (CR) 6/11, partial remission (PR) 3/11) with well-tolerated toxicity (Zhang et al., 2016a). This promising finding provides an effective alternative to address the challenge of antigen escape in anti-CD19 CAR-T cell therapy (Maude et al., 2014a; Davila et al., 2014; Lee et al., 2015a; Turtle et al., 2016a) by using CD19/CD20 bi-specific CAR, which has been proven by Eugenia et al. in a preclinical study (Zah et al., 2016). Other promising B cell lineage of antigens for CAR-T cell therapy in B cell malignancies such as CD22, inactive tyrosine-protein kinase transmembrane receptor (ROR1), and the immunoglobulin kappa chain (Igκ) are still undergoing clinical testing without results reported yet (Jackson et al., 2016). Here we review clinical results from trials investigating CAR-T cells in B-ALL, B-NHL, chronic lymphocytic leukemia (CLL), and Hodgkin’s lymphoma (HL) (Table 1).

**Table 1:** Summary of second-generation CAR-T trials for the treatment of B-cell malignancies

Disease	Treating institute	Patient populations	Target	Construct (scFv-Hinge-TM-CD-SD)	Gene transfer method	Conditioning therapy
B-ALL	MSKCC (Davila et al., 2014; Brentjens et al. 2011, 2013; Park et al. 2014, 2015)	Adult 44 43 evaluable for response	CD19	SJ25C1-CD28- CD3ζ	Retrovirus	Cy or Cy/Flu
	Upenn (Maude et al. 2014a; Grupp et al. 2013, 2015)	Pediatric and young adult 53	CD19	FMC63-CD8α-4-1BB-CD3 ζ	Lentivirus	Investigator’s choice 48/53 pts received
	NCI (Lee et al. 2015a, b)	Young adult 38	CD19	FMC63-CD28- CD3ζ	Retrovirus	27 Cy/Flu (Cy 900 mg/m² × 1, Flu 25 mg/m² × 3) 3 HD Cy/Flu (Cy 1200 mg/m² × 2, Flu 30 mg/m² × 4) 6 FLAG 2 IE
	FHCRC (Turtle et al. 2016a)	Adult 30 29 evaluable for response	CD19	FMC63-IgG4 CD28-4-1BB-CD3 ζ	Lentivirus	2 Cy/E (Cy 2–3 g/m² , etoposide 100 mg/m² × 3) 11 Cy (Cy 2–4 g/m²) 12 Cy/Flu3 (Cy 60 mg/kg × 1, Flu 25 mg/m² × 3) 5 Cy/Flu5 (Cy 60 mg/kg × 1 and Flu 25 mg/m² × 5)
B-NHL	NCI (Lee et al. 2015a; Kochenderfer et al. 2010, 2012, 2015, 2016)	Adult 39 29 DLBCL 6 FL 4 other histories	CD19	FMC63-CD28- CD3ζ	Retrovirus	Cy 120 or 60 mg/kg, Flu 25 mg/m^2^ × 5 Cy 300 or 500 mg/kg × 3 , Flu 30 mg/m^2^ × 3
	Upenn (Schuster et al. 2015)	Adult 24 13 DLBCL 9 FL 2 MCL	CD19	FMC63-CD8α-4-1BB-CD3 ζ	Lentivirus	Investigator’s choice
	FHCRC (Turtle et al. 2016b)	Adult 32 22 DLBCL 6 FL 4 MCL	CD19	FMC63-IgG4- CD28-4-1BB-CD3 ζ	Lentivirus	12 Cy or Cy/E (Cy 2–4 g/m^2^ +/− etoposide 100–200 mg/m^2^ × 3) 20 Cy/Flu (Cy 60 mg/kg; Flu 25 mg/m^2^ × 3–5)
	PLAGH (Wang et al. 2014)	Adult 7 DLBCL 6 evaluable for response	CD20	HB-9645-CD8α-4-1BB-CD3 ζ	Lentivirus	Investigator’s choice
	PLAGH (Zhang et al. 2016a)	Adult 11 8 DLBCL 1 FL 1 MCL 1 PCMZL	CD20	HB-9645-CD8α-4-1BB-CD3 ζ	Lentivirus	Investigator’s choice
CLL	Upenn (Kalos et al. 2011; Porter et al. 2015)	Adult 14	CD19	FMC63-CD8α-4-1BB-CD3 ζ	Lentivirus	6 Bendamustine 3 Cy/Flu 5 Pentostatin/Cy
	Upenn (Porter et al. 2016)	Adult Stage 1 28 24 evaluable for response Stage 2 21 17 evaluable for response	CD19	FMC63-CD8α-4-1BB-CD3 ζ	Lentivirus	N/A
HL	BCM (Ramos et al. 2015)	Adult 9 7 HL 2 ALCL	CD30	HRS3-IgG1-CD28- CD3ζ	Retrovirus	None
	PLAGH (Wang et al. 2017a)	Adult 18 17 HL 1 ALCL	CD30	BerH2-CD8α-4-1BB-CD3 ζ	Lentivirus	Investigator’s choice

Disease	Infused CAR-T cell dose	Responses observed	Persistence of CAR-T cells	Reported relapse	post-CAR-T cell allo-HSCT
B-ALL	1–3 × 10^6^/kg	CR: 36/43 (84%) (MRD- in 29/35, MRD analysis was performed in 35 of 36 CR pts) Median OS, 8.5 mo (all pts), 10.8 mo (MRD-CR pts). 7 pts remaining disease-free beyond 1 year up to 45 mo	Peaked within 1–2 wks, undetectable by 2 to 3 mo	14 relapse, with 2 CD19-relapse	12
	1–17.4 × 10^6^/kg	CR: 50/53 (94%) (MRD- in 45/50. 2 pts becoming MRD- by 3 mo with no further therapy) At 12 mo, 45% EFS, 44% RFS, 78% OS	B cell aplasia, 3–39 mo in pts with ongoing responses	20 relapse, with 13 CD19- relapse	6 (DLI or HSCT)
	1 × 10^6^/kg (32) 3 × 10^6^/kg (4) 28000/kg (1) 480000/kg (1)	CR: 23/38 (61%) (MRD- in 20/23) Median LFS, 17.7 mo with 45.5% probability of LFS beginning at 18 mo	Undetectable by day 68	2 CD19- relapse	17
	2 × 10^5^/kg (13) 2 × 10^6^/kg (15) 2 × 10^7^/kg (2) (1:1 CD4^+^:CD8^+^)	CR: 27/29 (93%) by flow cytometry (MRD- in 25/27. 1 pt becoming MRD- by d 83 with no further therapy) The median follow-up for Cy/Flu pts who were alive and in CR (12/17) was 300 days	5 pts had detectable CAR-T cells in the blood by Q-PCR (>10 copies/μg DNA) by the day 100 after cell infusion	9 relapse with 2 CD19- relapse	A few, but no detailed data available
B-NHL	1–5 × 10^6^/kg	ORR: 28/36 (78%) among evaluable pts with CR rate of 16/36 (44%) DLBCL: ORR/CR (19/27) 70%/(12/27) 44% FL: ORR/CR (5/5) 100%/(2/5) 40%	NA	NA	NA
	3.08–8.87 × 10^6^/kg	ORR: 15/22 (68%) among all evaluable pts at 3 mo, DLBCL 54% (7/13); FL 100% (7/7); MCL 50% (1/2) At the median follow-up 11.7 mo, 62% PFS (DLBCL 43%; FL 100%). For responders at median follow up, response duration is 83% for DLBCL and 100% for FL	NA	NA	NA
	2 × 10^5^/kg (5) 2 × 10^6^/kg (18) 2 × 10^7^/kg (9) (1:1 CD4^+^:CD8^+^)	ORR: 19/30 (63%) among all evaluable pts with CR rate of 10/30 (33%) DLBCL: ORR/CR (14/21) 67%/(8/21) 38% FL: ORR/CR (4/5) 80%/(2/5) 40% MCL: ORR/CR (1/4) 25%/(0/4) 0% For the pts received ≤2 × 10^6^ CAR-T cells/kg, the median OS follow-up times for No Flu and Cy/Flu are 25 and 6.3 mo, respectively. The median PFS follow-up for Cy/Flu is 5.8 mo. The median PFS for No Flu is 1.5 mo	In the Cy/Flu group,16 of 18 pts had detectable CAR-T cells in the blood by Q-PCR (>10 copies/μg DNA) at the last follow-up (range, 34–349 days)	In the Cy/Flu group, 1 relapse	A few, but no detailed data available
	0.36–2.35 × 10^7^/kg	ORR: 4/6 (67%) among all evaluable pts with CR rate of (1/6) 17% Duration of response: PR: 3–6 mo CR: 14 mo+	PB: at least 4 wks with a higher CAR gene copy number (1000/μg DNA); Biopsy tissue: 10 wks	NA	NA
	0.41–1.46 × 10^7^/kg	ORR: 9/11 (82%) with CR rate of 6/11 (55%) DLBCL: ORR/CR 7/8 (88%)/4/8 (50%) Duration of response: CR: 4–27 mo (3 ongoing) PR: including an ongoing 13 mo PR Median PFS, 6 mo	Peaked at ~4 wks, persisted up to 12 wks in most of pts. 1 pt , persistence >2 years	5 CD20+ relapse	NA
CLL	0.14–11 × 10^8^	ORR: 8/14 (57%) with CR rate of 4/14 (29%) 3 of 4 pts achieving CR maintained response for 40 mo (range 28–53)	14–49 mo in the 4 pts achieving CR, CAR-T cells isolated from one of these pts almost 3 years post cell infusion retained functional	No relapse if pts achieved CR	NA
	Stage 1 5 × 10^7^ (13) 5 × 10^8^ (11) Stage 2 5 × 10^8^ (17)	Stage 1 High dose group, ORR: 6/11 (55%) with CR rate of 4/11 (36%); low dose group, ORR: 4/13 (31%) with CR rate of 1/13 (8%) Stage 2 ORR: 9/17 (53%) with CR rate of 6/17 (35%) 5 pts remain in CR with median follow-up 26 mo (range 5–34)	NA	1 CD19− relapse	NA
HL	2 × 10^7^/m^2^ (2) 1 × 10^8^/m^2^ (2) 2 × 10^8^/m^2^ (5)	1 CR,1 PR,4 SD (persisting for 1.5–8 mo)	Peaked at 1 wk, decreased to near background by 4 wks	NA	NA
	1.1–2.1 × 10^7^/kg	7 PR (persisting for 2–9 mo, and 3 remissions are ongoing) 6 SD Median PFS, 6 mo	Peaked at ~1 wk ,and decreased to the baseline level by 4–8 wks, while in which time relatively higher numbers in biopsy tissues were detected	NA	NA

Abbreviations: MSKCC, Memorial Sloan Kettering Cancer Center; Upenn, University of Pennsylvania; NCI, National Cancer Institute; FHCRC, Fred Hutchinson Cancer Research Center; PLAGH, Chinese PLA General Hospital; BCM, Baylor College of Medicine; scFv, single chain variable fragment; B-ALL, B cell acute lymphoblastic leukemia; B-NHL, B cell non -Hodgkin’s lymphoma; CLL, chronic lymphocytic leukemia; HL, Hodgkin’s lymphoma; DLBCL, diffuse large B cell lymphoma; FL, follicular lymphoma; MCL, mantle cell lymphoma; PCMZL, primary cutaneous marginal zone lymphoma; ALCL, anaplastic large cell lymphoma; Cy, cyclophosphamide; Flu, fludarabine; FIAG, fludarabine+Ara-c+G-CSF; IE, ifosfamide/etoposide; HD, high dose; ORR, objective remission rate; CR, complete remission; PR, partial remission; SD, stable disease; MRD, minimal residual disease; MRD-CR, MRD-negative CR; EFS, event-free survival; RFS, relapse-free survival; OS, overall survival; PFS, Progression-free survival; PB, peripheral blood; CAR-T, chimeric antigen receptor modified T cell; allo-HSCT, allogeneic-hematopoietic stem cell transplantation; DLI, donor lymphocyte infusion; NA, not applicable; pts, patients; mo, month(s); wks, weeks; +, ongoing

### B cell acute lymphoblastic leukemia

Up to now, CAR-T cell therapy has been most effective in patients with B-ALL as significant CR rates of 70%–94% were observed even in a post-allogeneic-hematopoietic stem cell transplantation (allo-HSCT) setting (Maude et al., 2014a; Davila et al., 2014; Lee et al., 2015a; Turtle et al., 2016a). Based on the promising results of the initial phase I trials, several pivotal phase II clinical trials evaluating CAR-T cell therapy for B-ALL are underway (US National Library of Science, 2016a, b, c, d, e). However, it is difficult to draw a conclusion about which is better according to the clinical response rate as these trials varied significantly in the key factors that determine the final efficacy, including CAR structure, preconditioning regimen, infused T cell product, T cell dose, etc. Even relapsed or refractory B-ALL (R/R B-ALL) was adopted by almost all institutions for patient selection, patient age, risk features, prior treatment history, and degree of tumor burden at the time of CAR-T cell infusion has been widely discrepant, which led to the trials being more heterogeneous.

MSKCC was the first to publish results of CD19-targeted CAR for adults with R/R B-ALL (NCT01044069) (Brentjens et al., 2011). Updated results of this study were reported by Brentjens et al. (2013), where all five adult patients treated with anti-CD19 28ζ CAR-T (19-28ζ CAR-T) cells at a dose of 1.5–3 × 10^6^ 19-28ζ CAR-T cells/kg achieved minimal residual disease(MRD)-negative CR(MRD-CR). Clinical efficacy of this approach has been confirmed and reached a climax in the follow-up study of an additional 11 adult patients treated with R/R B-ALL (Davila et al., 2014). A total of 14 of 16 (88%) patients achieved morphologic CR or CR with incomplete blood count recovery (CRi), and 12 of the 16 (75%) were classified as MRD-negative following CAR-T cell infusion. However, two patients already had been rendered MRD-negative by salvage therapy prior to CAR-T cell infusion, potentially confounding the role of 19-28ζ CAR-T cells. 19-28ζ CAR-T cell expansion *in vivo* peaked within 12 weeks and persisted for 2–3 months post-infusion in most patients, supplying a window of time following transplant; hence researchers defined the 19-28ζ CAR-T cell therapy as a “bridge” to transplant. This study also first defined the diagnostic criteria for severe cytokine release syndrome (sCRS) secondary to CAR-T cell infusion, and identified C-reactive protein (CRP) as a potential laboratory indicator for CRS severity that could be used as a surrogate for cytokines. The long-term outcome containing survival data for this study was demonstrated in a larger cohort of 22 evaluable patients; the median overall survival (OS) is 9 months and 5 patients have relapsed, including 1 with CD19-negative disease (Park et al., 2014). At the 2015 annual meeting of the American Society of Hematology (ASH), they updated their experience in 44 adults with 43 evaluable patients who received lymphodepleting chemotherapy followed 2 days later by 1–3 × 10^6^ 19-28ζ CAR-T cells/kg (Park et al., 2015). The potent anti-tumor efficacy of 19-28ζ CAR-T cells in adults with R/R B-ALL has been confirmed as a similar CR rate of 84% (36/43) and MRD-CR rate of 67% (29/43) was observed in this larger cohort study. Median overall survival (OS) of all patients and those who achieved MRD-CR was 8.5 months and 10.8 months, respectively. Therefore, the researchers concluded that MRD negativity following the 19-28ζ CAR-T cell treatment was highly predictive of survival. It is worth noting that allo-HSCT post-CAR-T cell infusion had no significant impact on the survival of the patients who achieved CR as the OS at 6 months is similar (70% vs. 64%) between the patients who underwent post-CAR allo-HSCT and those who did not. This finding is amazing in the context of the persistence of 19-28ζ CAR cells is 2–3 months post-infusion as reported previously (Davila et al., 2014), which warrants further investigation.

On the basis of an initial successful experience of using CD19-specific BBζ CAR transduced T cells (termed CTL019) to treat three patients with CLL (NCT01029366) (Porter et al., 2011; Kalos et al., 2011), researchers at the Children’s Hospital of Philadelphia and the University of Pennsylvania (CHOP/UPenn) conducted a phase I trial to investigate CTL019 cells for children with R/R B-ALL (NCT01626495) and presented a case report on the first two patients in 2013 (Grupp et al., 2013). CR was observed in both patients and was ongoing in one patient at 11 months after treatment (Ongoing CR at 3 years post-CTL019 cell infusion has been described (Tasian and Gardner 2015)), while the other patient experienced a CD19-negative relapse 2 months post-CAR-T cell infusion. Although only two patients were reported, it made sense to CAR-T cell therapy development as it not only provided an effective approach to the management of sCRS by incorporating tocilizumab (a recombinant humanized monoclonal antibody against interleukin-6 receptor (IL-6R)) without compromising efficacy, but also highlighted the significant threat to a successful CAR regimen, namely, tumor antigen loss escape. The updated outcomes of an expanded cohort of 25 children and 5 adults with R/R B-ALL who received CTL019 cells at a dose of 0.76–20.6 × 10^6^ CTL019 cells/kg following the investigator’s choice lymphodepleting regimen were reported by Maude et al. (2014a). Morphologic CR was achieved in 27 patients (90%), including 2 with blinatumomab-refractory disease and 15 who had undergone stem-cell transplantation; 22 of 27 (81%) patients achieved MRD-CR. Seven patients achieving CR subsequently experienced relapse (3 with CD19-negative disease) between 6 weeks and 8.5 months after the infusion of CTL019 cells. However, it was noted that prolonged persistence of CTL019 cells and B cell aplasia for as long as 2 years was seen in this study, implying CTL019 cells could be proposed as a potential treatment alternative for patients who are ineligible for stem-cell transplantation. The investigators also reported the outcomes and longer follow-up of the first 53 children or young adults with R/R B-ALL treated with a median of 4.3 × 10^6^ CTL019 cells/kg (1–17.4 × 10^6^ cells /kg) (Grupp et al., 2015). At day 28 post CAR-T cell infusion, 50 patients (94%) achieved morphologic CR, including 45 patients who achieved MRD-CR measured by clinical flow cytometry. Intriguingly, two additional patients in morphologic CR at day 28 achieved MRD-CR by 3 months without further therapy. However, 12 of 53 evaluable patients had already been MRD-negative attributed to lymphodepleting chemotherapy at the time of CTL019 cell infusion, which is similar to the aforementioned MSKCC report (Davila et al., 2014). Twenty of fifty patients with CR at day 28 had subsequently relapsed (relapse-free survival is 44% at 12 months), thirteen of whom experienced CD19-negative disease relapse, which should be the most frequent CD19-negative relapse in the available data to date (Maude et al., 2014a; Davila et al., 2014; Lee et al., 2015a, b; Turtle et al., 2016a; Park et al., 2015). They also believed rapid loss of CTL019 cells (prior to 3 months) was associated with a high risk of CD19+ relapse.

The 2 aforementioned institutions all infused CAR-T cells at a relatively broad dose range, while a fixed dose of either 1 × 10^6^ or 3 × 10^6^ CAR-T cells/kg was employed in a phase I dose escalation study (NCT01593696) performed at the NCI to investigate anti-CD19 28ζ CAR-T cells for children and young adults with R/R B-ALL (Lee et al., 2015a). Twenty patients with R/R B-ALL and one patient with diffuse large B cell lymphoma (DLBCL) were treated; the maximum tolerated dose (MTD) for the entire cohort was defined as 1 × 10^6^ CAR-T cells/kg by using a 3 + 3 dose-escalation schema. Fourteen of twenty (70%) patients with B-ALL achieved morphologic CR, with twelve achieving MRD-CR. No CAR-T cells were detected at day 68 post-CAR-T cell infusion in any patient; the persistence of CAR-T cells is similar to those observed by investigators at MSKCC. Ten of twelve patients who were MRD-negative went on to HSCT and all remained disease-free at a median follow-up of 10 months. Therefore, the researchers concluded that anti-CD19 CAR-T cell therapy was an effective bridge to HSCT in patients with refractory B-ALL, which simultaneously explained why the researchers believed that long-term persistence was not necessary to induce meaningful anti-tumor effects. However, the other two patients who did not receive allo-HSCT experienced CD19-negative disease relapse at 3 and 5 months. This study also provided the first evidence that anti-CD19 CAR-T cell could eradicate leukemia in cerebrospinal fluid without long-term toxicity. Additional 18 children or young adults with R/R B-ALL were treated with a selected dose of 1 × 10^6^ CAR-T cells/kg, and updated experiences with the first 38 patients were reported at ASH 2015 (Lee et al., 2015b). Thirty-eight patients across both cohorts showed a morphological CR and MRD-CR rate of 61% and 53%, 13/16 (81%) of low-burden patients had a morphological CR, while 10/22 (45%) of high-burden patients attained a morphological CR. Of the 20 patients achieving an MRD-CR, the median leukemia-free survival (LFS) was 17.7 months, with a 45.5% probability of LFS beginning at 18 months.

In contrast to the strategy of infusion of a mixture of T cell products without preselecting, which is well accepted and widely used by most institutions (Gill and June, 2015), investigators at FHCRC believed that preselecting specific T cell subsets and using defined formulations would be informative for enhancing the potency and reproducibility of cancer immunotherapy, according to their preclinical data (Wang et al., 2011; Terakura et al., 2012; Sommermeyer et al., 2016). Therefore, Turtle et al. conducted a phase I/II trial (NCT01865617) evaluating CD19-targeted BBζ CAR-T cell therapy for advanced CLL, ALL, and lymphoma, in which the T cell products were formulated in a defined 1:1 ratio of CD8^+^ and CD4^+^ T cell subsets (Turtle et al., 2016a). Promising preliminary results were achieved in 29 evaluable adults with R/R B-ALL who received 2 × 10^5^, 2 × 10^6^, or 2 × 10^7^ CAR-T cells/kg in a defined 1:1 ration of CD4:CD8 composition. 27 of 29 patients (93%) achieved bone marrow (BM) remission, as leukemia was undetectable by high-resolution flow cytometry. Investigators observed a marked increase in CAR-T cell expansion and persistence in 17 patients who received cyclophosphamide and fludarabine (Cy/Flu) lymphodepletion compared with 12 patients who received lymphodepletion with Cy alone or with etoposide. As a consequence of these enhancements, an improvement in overall and disease-free survival also was observed in the Cy/Flu cohort, wherein only 2 of 17 (12%) patients relapsed (1 CD19+ relapse, 1 CD19− relapse) post-CAR-T cell infusion. In contrast, 7 of 12 patients (58%) relapsed (6 CD19+ relapses, 1 CD19− relapse) post-CAR-T cell infusion in the cohort without Flu. While these data are encouraging, additional patient accrual and longer follow-up periods are required. Moreover, researchers identified a T cell-mediated anti-CAR immune response specific for murine scFv epitopes in the patients in whom CAR-T cells failed to persist after the second infusion. This result is similar to the finding recorded in detail in the other CAR trials (Lamers et al., 2011; Maus et al., 2013; Jensen et al., 2010), highlighting the immunogenicity of murine CAR, especially when it is administered using an intermittent dosing schedule.

### B cell non-Hodgkin’s lymphoma

To date, successful experience in patients with B-NHL still mainly generated from the clinical trial using anti-CD19 CAR-T cells; however, CD20-targeted CAR-T cells have also been employed (Zhang et al., 2016a; Till et al., 2008, 2012; Jensen et al., 2010) and have demonstrated potential therapeutic value (Zhang et al., 2016a). Patients with DLBCL and follicular lymphoma (FL) represent of the majority in those clinical trials (Batlevi et al., 2016). The clinical efficacy of CAR-T cell therapy for patients with B-NHL is not as robust as those with R/R B-ALL, for reasons that are not well-defined, but disease-driven depletion of early lineage cells in lymphoma may be a contributing factor (Singh et al., 2016).

Publication of the early clinical trials to evaluate first-generation CAR-T cell therapy for B-NHL occurred in 2008 (Till et al., 2008) and 2010 (Jensen et al., 2010), but there was no evidence of clinical benefit. The researchers at BCM have attempted to simultaneously infuse first- and second-generation CAR-T cells targeting CD19 into patients with active FL or DLBCL; still no clinical benefit was observed, but CAR including CD28 costimulatory domains led to enhanced *in vivo* expansion and the persistence of CAR-T cells has been demonstrated (Savoldo et al., 2011).

So far, investigators at the NCI have presented the largest data series from clinical trials investigating CD19-targeted CAR-T cells for B-NHL; a cumulative 36 evaluable patients including 27 patients with various DLBCL show an ORR and CR rate of 78% and 44%. In 2010, researchers presented the first PR lasting 32 weeks in a patient with advanced FL who received lymphodepleting chemotherapy followed by an infusion of anti-CD19 28ζ CAR-T cells (NCT00924326) (Kochenderfer et al., 2010). An updated outcome of this trial in four patients with B-NHL and four patients with CLL was reported by Kochenderfer et al. (2012). All the 3 evaluable patients with advanced, progressive B-NHL (2 FL, 1 splenic marginal zone lymphoma (SMZL)) who received conditioning chemotherapy followed by an infusion of anti-CD19 28ζ CAR-T cells and a course of IL-2 obtained PR. Durations of response ranged from 8 to 18 months, and two remissions were ongoing. Of note, the first patient obtaining PR previously reported (Kochenderfer et al., 2010) developed progressive CD19+ lymphoma 32 weeks after his first infusion of anti-CD19 28ζ CAR-T cells, whereas B cell dysplasia lasted 39 weeks and 36 weeks after the first CAR-T cell infusion in the peripheral blood (PB) and BM, respectively. This patient was retreated on the same protocol and was in an 18-month ongoing PR after the second treatment (Kochenderfer et al., 2012; Kochenderfer and Rosenberg, 2011). More impressive results of this study were observed in a larger cohort of 15 patients with B-NHL (9 DLBCL, containing 4 primary mediastinal B cell lymphoma, 1 SMZL, and 1 low-grade NHL) and 4 patients with CLL who were treated with lymphodepleting chemotherapy followed by infusion of anti-CD19 28ζ CAR-T cells at a dose of 1–5 × 10^6^ CAR-T cells/kg without IL-2 (Kochenderfer et al., 2015). Lymphodepleting chemotherapy was Cy at a total dose of either 120 or 60 mg/kg, followed by five daily doses of Flu 25 mg/m^2^. Of the seven evaluable patients with DLBCL, four obtained CR, two obtained PR; in three of these four CR are ongoing, with durations ranging from 9 to 22 months. The patients with SMZL were previously treated on their anti-CD19 CAR-T cell protocol and obtained a PR lasting 12 weeks (Kochenderfer et al., 2012), then were retreated with the same regimen and obtained a PR with an ongoing response of 23 months as of the time of writing. The most troublesome toxicities were hypotension and neurologic toxicities that can be resolved within 3 weeks after cell infusion. One patient died 16 days after cell infusion from an undetermined reason. In 2016, at the American Society of Clinical Oncology (ASCO) annual meeting, researchers presented an updated outcome of this study, wherein 22 patients with B-NHL were treated with a low dose of FC lymphodepleting chemotherapy regimen of Cy either 300 or 500 mg/kg daily for 3 days and Flu 30 mg/m^2^ daily for 3 days on the same days as Cy followed by a single infusion of anti-CD19 28ζ CAR-T cells (Kochenderfer et al., 2016). Of the 19 patients treated with various subtypes of DLBCL, 8 had CR, 5 had PR, 2 achieved stable disease (SD), and the other 3 (1 mantle cell lymphoma (MCL), 2 FL) obtained CR. Durations of response ranged from 1 to 20 months, and 10 remissions were ongoing. However, only 4 of all 22 treated patients had either chemotherapy refractory lymphoma or lymphoma that had relapsed after autologous stem cell transplant, undoubtedly comprising the significant efficacy of this regimen. Neurologic toxicities were still the most prominent toxicities; fever and hypotension were also observed in some patients. Intriguingly, the researchers found that patients obtaining CR or PR had higher peak blood CAR^+^ cell levels than patients experiencing SD or PD. This group also reported the result of a trial (NCT01087294) to evaluate donor-derived CD19-targeted 28ζ CAR-T cells without prior lymphodepleting chemotherapy for patients with B-NHL or CLL in whom tumor lesions persisted after allo-HSCT and standard donor lymphocyte infusions (DLIs) (Kochenderfer et al., 2013). Of the 10 treated patients (2 DLBCL, 4 MCL, and 4 CLL), only 1 patient with CLL obtained a 9-month ongoing CR; 2 patients with MCL experienced PR. This less encouraging outcome could be attribute to no prior lymphodepleting chemotherapy, resulting in less than 1 month persistence of CAR-T cells. No graft-versus-host disease (GVHD) was observed in any of the patients. Toxicities included transient hypotension and fever. Updated results of the first 20 patients with B cell malignancies (5 CLL, 10 B-NHL, and 5 B-ALL) that progressed after allo-HSCT who received allogeneic T cells transduced with CAR targeting CD19 were reported by Brudno et al. (2016). An ORR of 40% with 30% CR was observed among 20 treated patients. Of the 10 treated patients with B-NHL, 1 CR and 1 PR were achieved. None of the treated patients has experienced new-onset acute GVHD post-CAR-T cell infusion.

Investigators at Upenn also updated their preliminary results of a phase IIa trial (NCT02030834) evaluating CTL019 cells for patients with relapsed or refractory lymphomas (Schuster et al., 2015). A total of 38 patients (21 DLBCL, 14 FL, and 3 MCL) were enrolled, and eventually 24 patients (13 DLBCL, 9 FL, and 2 MCL) were treated with physician’s choice conditioning therapy followed by a single infusion of CTL019 at a median dose of 5.84 × 10^6^ CAR^+^ T cells/kg (range: 3.08–8.87 × 10^6^ CAR^+^ T cells/kg). A 68% (15/22) ORR was achieved at 3 months post CTL019 infusion in the 22 evaluable patients (13 DLBCL, 7 FL, and 2 MCL). Progression-free survival (PFS) at the median follow-up of 11.7 months was 62% (DLBCL 43%, FL 100%), at which time the response duration was 83% for DLBCL and 100% for FL. However, more detailed efficacy data such as CR rate and CAR-T cell persistence *in vivo* were not presented in the abstract. A total of 16 of 24 (67%) treated patients developed grade 2–4 CRS with 1 grade 4 CRS. Three patients developed neurologic toxicity, including transient delirium (1 grade 2, 1 grade 3) and 1 possibly related grade 5 encephalopathy.

Differing from the above-mentioned notion that defines CAR-T cell therapy as a treatment alternative for patients with R/R B-NHL, investigators at MSKCC tried to evaluate whether those patients who have been treated with high-dose therapy and autologous stem cell transplant (HDT-ASCT) can benefit from CAR-T cell consolidation. At the 2015 ASCO annual meeting, researchers reported the safety data of a phase I dose-escalation study (NCT01840566) in 8 patients with poor-risk R/R aggressive B-NHL who received BEAM-conditioned HDT-ASCT followed by infusion of anti-CD19 28ζ CAR-T cells at 1 of 3 dose levels (5 × 10^6^, 1 × 10^7^ or 2 × 10^7^ CAR^+^ T cells/kg) at days +2 and +3 (Sauter et al., 2015). Besides one patient who received dose level 2 (1 × 10^7^ CAR^+^ T cells/kg), others were treated at dose level 1 (5 × 10^6^ CAR^+^ T cells/kg). Half of the patients had CRS, which could be well managed with tocilizumab and/or corticosteroids. One patient died from non-relapse mortality (NRM) of mucormycosis pneumonia at day 38 after HDT-ASCT. Five of eight patients with PET(+) PR prior to HDT-ASCT obtained CR with duration ranging from 10 to 18 months post-HDT-ASCT, but whether CAR-T cell therapy contributed to this higher CR rate and longer duration of response still needs further exploration. Investigators at FHCRC reported the outcome of the aforementioned phase I/II trial (NCT01865617) (Turtle et al., 2016a) in 32 patients with R/R B-NHL (22 DLBCL, 6 FL, and 4 MCL) who were treated with the same protocol for patients with B-ALL (Turtle et al., 2016b). Twelve and twenty patients with B-NHL received Cy-based conditioning regimens without Flu or with Flu, respectively. A 50% ORR with 8% CR rate was obtained among 12 evaluable patients in the Cy-based without Flu group, whereas a 72% ORR with 50% CR rate was observed in the Cy/Flu group (18 evaluable patients). Researchers again observed a CD8-mediated immune response as observed in B-ALL (Turtle et al., 2016a) due to CAR transgene immunogenicity, leading to no significant T cell expansion or clinical responses in five of five patients who received a second reinfusion of CAR-T cells. Investigators believed that this finding provided one potential mechanism for the loss of CAR-T cells observed in other trials and concluded that Cy/Flu could minimize the substantial cellular immune response against CAR. sCRS and grade ≥3 neurotoxicity were observed in 13% and 28% of all patients, respectively. Of note, no patient treated at all three dose levels experienced sCRS in the Cy-based without Flu group, whereas three patients experienced sCRS and four patients developed grade ≥3 neurotoxicity among six patients who received 2 × 10^7^ CAR^+^ T cells/kg following Cy/Flu, implying the toxicities might be related to the cell dose, especially in the context of that the Cy/Flu conditioning regimen was used. Peak IL-6, interleukin-15 (IL-15), interferon-γ (IFN- γ), and interleukin-10 (IL-10), concentrations on day 1 after CAR-T cell infusion have been determined to have a strong correlation with subsequent sCRS and neurotoxicity; nonetheless, whether those serum biomarkers can be used as accurate predictive biomarkers for sCRS and neurotoxicity remains to be elucidated.

Investigators at PLAGH presented the preliminary result of the study (NCT01735604) to evaluate anti-CD20 BBζ CAR-T cells (referred as CART-20) for R/R B-NHL in 2014 (Wang et al., 2014). Seven heavily pretreated patients with refractory advanced CD20+ DLBCL were treated with CART-20 cells at a dose of 0.36–2.35 × 10^7^ CAR^+^ T cells/kg alone or following physician’s choice debulking chemotherapy in order to alleviate tumor load as well as conditioning. Four of six evaluable patients had bulk tumor burdens defined as lesion(s) with the longest diameter greater than 5 cm or more than three lesions, and 3 of whom achieved PR with a duration ranging from 3 to 6 months by infusion of CART-20 cells. Among the other two patients with no bulky tumors, a 14-month ongoing CR occurred after CART-20 cells infusion alone. CART-20 cells in PB could persist for at least 4 weeks in most patients with a higher CAR gene copy number (1000/μg DNA), which also could be detected in biopsy tissues derived from three of six evaluable patients even after 10 weeks of cell infusion. Correspondingly, a decrease of the CD20^+^ B cell count in PB was observed, which could be attributed to the on-target/off-tumor recognition of CART-20 cells. Six of the seven treated patients developed delayed toxicities mainly due to the cytokine elevation related to CART-20 cells 3–8 weeks post-CART-20 cells infusion, except for a grade 4 acute alimentary tract hemorrhage resulting in death. An impressive result with 82% ORR with a 55% CR rate was shown in the phase IIa study of 11 patients with refractory or relapsed CD20^+^ B-NHL (8 DLBCL, 1 FL, 1 MCL, and 1 primary cutaneous marginal zone lymphoma (PCMZL)) who were treated with the same protocol (Zhang et al., 2016a). Of eight patients with DLBCL, four obtained CR with a duration ranging from 4 to 27 months, three remissions were ongoing, and three achieved PR, including one 13-month ongoing PR. Two CR both lasting 5 months were achieved among the other three patients with indolent B-NHL. The median PFS was 6 months. Five patients who had response relapsed with CD20 positive between 60 days and 6 months after infusion of CART-20 cells when the CAR gene copy number declined to the near lowest value as well as polyclonal B cells recovered from aplasia, illustrating an inverse correlation between CAR molecule levels in PB and the CD20^+^ target cell. No grade 4 toxicities and CRS developed, which mainly should be attributed to the fact that no patient with defined bulky tumors was enrolled, as the lessons drawn from the phase I study that high tumor burden increased the risks of severe toxicities.

### Chronic lymphocytic leukemia

The exploration of CAR-T cells targeting CD19 for patients with CLL is earlier than B-ALL; however, less mature data have been reported. Moreover, although all express CD19, it appears that CLL has a lower response rate than B-ALL, with an ORR of 62% across publications by 2014 (Zhang et al., 2015). *In vivo* disease-intrinsic mechanisms such as defects in the circulating T cells of CLL patients and/or the inhibitory microenvironment associated with this often bulky disease may contribute to this relative paucity of response (Pegram et al., 2015; Kalos 2016; Khalil et al., 2016).

The largest cohort of CD19-targeted CAR-T cell therapy for CLL has been reported by investigators at Upenn. As of now, CTL019 has treated more than 45 patients with relapsed and refractory CLL (R/R CLL) and has shown an ORR of ~45% (Maude et al., 2015a). Researchers at Upenn in their pilot clinical trial (NCT01029366) first demonstrated that CTL019 could induce dramatic antitumor response for patients with advanced, chemotherapy-resistant CLL, where two ongoing CR and one PR were achieved in the three treated patients (Kalos et al., 2011). Mature results from this pilot clinical trial using CTL019 treatment of 14 patients with R/R CLL at a dose of 0.14–11 × 10^8^ CTL019 cells (median, 1.6 × 10^8^ cells) were presented by Porter et al. (2015). The ORR was 8 of 14 (57%), with 4 CR and 4 PR including the aforementioned 3 outcomes (Kalos et al., 2011). Three of the four patients achieving CR maintained this response for 40 months (range 28–53); the other patient died from infection while in CR 21 months after treatment. However, a relatively shorter duration of response (range 5–13 months) was observed in all four patients who attained PR, which was correlated with the *in vivo* expansion and persistence of the CAR-T cells. Significantly, CTL019 cells could be detected in the first two patients achieving CR 4 years post CTL019 cells infusion, and CTL019 cells isolated from one who was almost 3 years post-CTL019 cells infusion remained functional, highlighting that CAR-T cells could persist over the long term as memory cells and continually provide immunosurveillance and prevent relapse. This finding increases confidence that CAR-T cell therapy could be defined as a stand-alone therapy, at least for R/R CLL. A phase II dose optimization study (NCT01747486) was opened subsequently, in which 28 patients with R/R CLL were randomized to receive either 5 × 10^8^ or 5 × 10^7^ CTL019 cells following a preconditioning regimen (Porter et al., 2016). This ongoing trial confirmed the initial outcomes of the pilot study, albeit the ORR was slightly lower at 42% (5 CR, 5 PR) among 24 evaluable patients (11 high dose, 13 low dose). Moreover, the researchers identified 5 × 10^8^ CTL019 cells as the optimal dose of CTL019 in patients with R/R CLL on account of a relatively high ORR but with similar toxicity shown in the high-dose cohort compared with the low-dose cohort. Twenty-one patients with R/R CLL have been subsequently treated with the selected dose, and 9 patients had a response with 6 CR among the 17 evaluable patients, including 11 who had been treated at stage 1. Remissions were ongoing in five of six patients achieving CR at a median follow-up of 26 months (range 5–34); the other progressed with CD19 negative disease.

Other institutions, including MSKCC, NCI, and FHCRC, also have conducted initial clinical trials to evaluate autologous CD19-targeted CAR-T cells for R/R CLL. Across all three centers in 30 patients with R/R CLL (Geyer and Brentjens 2016), there was an ORR and CR rate of 53 and 30%, 31 and 13% at MSKCC (Brentjens et al., 2011; Geyer et al., 2016), 88 and 50% at NCI (Kochenderfer et al., 2012, 2015), and 67 and 50% at FHCRC (Turtle et al., 2015).

### Hodgkin’s lymphoma

Although Hodgkin’s lymphoma (HL) is a B-cell derived cancer, the tumor cells of HL- Hodgkin and Reed-Sternberg (HRS) cells have lost the B cell phenotypes such as CD19, CD20, or CD22, and are instead characterized by bright, uniform expression of CD30, which is also shared by a small population of activated T cells (Kuppers et al., 2012). Antibody-drug conjugate brentuximab vedotin (BV) directed to CD30 has been approved for treatment of relapsed HL as an objective antitumor response with a well-tolerated toxicity (Younes et al., 2010). Importantly, patients who relapse after prior BV appear to retain CD30 expression on HRS cells (Gill and June 2015). Another concern with targeting CD30 for CAR-T cell therapy in HL is that high concentrations of soluble CD30 have been found in patients with progressed HL, which may compete for CAR binding (Jackson et al., 2016); However, a preclinical study showed that this concern was unwarranted (Hombach et al., 1998). Taken together, it could make sense to develop a CD30-targeted CAR for HL.

Two trials evaluating anti-CD30 CAR-T cells for HL are ongoing at BCM (NCT01192464, NCT01316146). Ramos et al. reported (Ramos et al., 2015) the preliminary results of nine patients with lymphoma (7 HL, 2 anaplastic large cell lymphoma (ALCL)) who received 2 × 10^7^, 1 × 10^8^, or 2 × 10^8^ autologous CD30-specific CAR-T cells/m^2^ without a conditioning regimen. Eight of these patients had relapsed or progressed post-brentuximab treatment. At 6 weeks after treatment, one CR, one PR, and four SD were achieved among the nine treated patients, while persistence of CD30-specific CAR-T cell was limited as the molecular signal from CAR-T cells declined to near baseline by 4 weeks post-infusion. A dose of 2 × 10^8^ CD30-specific CAR-T cells/m^2^ was safe and associated with significant *in vivo* expansion compared to other dose cohorts. No adverse events (AEs) were observed, including CRS correlated with CAR-T cell infusion. The study also showed that the frequency of T cells responding to the virus remained unchanged in the CD30-specific CAR-T cell recipients, which implied fratricide that might have occurred as the transient expression of CD30 in activated T cells had not happened. A preclinical study to explore the risks of targeting CD30 by CAR-T cell therapy in humanized mice also confirmed what Ramos et al. observed as the research illustrated CAR-T cells targeting CD30 could confer a superior therapeutic index in the treatment of CD30^+^ malignancies, leaving healthy activated lymphocytes and hematopoietic stem and progenitor cells (HSPCs) unaffected (Hombach et al., 2016).

Publication of the first results of CD30-targeted CAR-T cells for patients with HL came from the investigators at PLAGH (NCT02259556) (Wang et al., 2017a). Eighteen heavily pretreated patients with lymphoma (17 HL, 1 primary cutaneous anaplastic large cell lymphoma) were enrolled, 15 of whom had a considerable burden of lymphoma characterized by multiple tumor lesions including extensive abnormal lymph node regions (range: 0–7) and extranodal disease involving the bone, lung, liver, pleura, mammary glands, kidney, and soft tissues. A median of 1.56 × 10^7^ CAR^+^ T cells/kg (range: 1.1–2.1 × 10^7^ cells/kg) were infused over 3–5 days following physician’s choice conditioning chemotherapy. Among the 18 treated patients, 7 patients achieved PR with durations ranging from 2 to 9 months (Three remissions were ongoing), and 6 had SD. Median PFS was 6 months, and the copy number of CAR transgenes in PB peaked about 1 week after infusion and decreased to the baseline level by 4–8 weeks in most patients, while in which time relatively higher numbers in biopsy tissues were detected, and a corresponding decrease of CD30^+^ tumor cells was observed in some patients, highlighting that CAR-T cells could traffic to tumor sites and remain functional. Importantly, patients appeared to benefit from second or multiple CAR-T cell infusions as ongoing responses were observed in most of patients who received a second CAR-T cell infusion and the decrease of tumor burden was more significant after the second CAR-T cell infusion compared to the first. It was noted that lymph nodes presented a better response than extranodal lesions; lung lesions were likely to be relatively poor. The infusion was well tolerated and no evidence of CRS occurred in all the treated patients, except two who experienced grade ≥3 toxicities.

## CAR-T CELL THERAPY FOR SOLID TUMORS

CAR-T cell therapy has shown enormous promise in B cell malignancies. However, this success has not yet extrapolated to solid tumors as they confer several challenges, especially for selecting appropriate targets. To date, there are only a few publications reporting clinical trials to evaluate second- or third-generation CAR-T cells in solid tumors by targeting human epidermal growth factor receptor-2 (HER2) (Morgan et al., 2010; Ahmed et al., 2015), mesothelin (MSLN) (Maus et al., 2013; Beatty et al., 2014), carcinoembryonic antigen (CEA) (Katz et al., 2015), and epidermal growth factor receptor (EGFR) (Feng et al., 2016). The efficacy is less encouraging, until recently, a significant clinical response with lower toxicities has been elicited in a patient with highly aggressive recurrent multifocal glioblastoma multiforme (GBM) who received both intracavitary and intraventricular administration of the interleukin-13 receptor alpha2 (IL13Rα2)-directed BBζ CAR-T cells (Brown et al., 2016), highlighting that CAR-T cell therapy could be useful for treating solid tumors by continuous optimization. Currently ongoing trials targeting solid tumors are listed in Table 2. In short, using CAR-T cells for solid tumors is still in a “proof-of-concept” stage, and feasibility and efficacy remain to be further established in clinical trials. Herein we review the preliminary outcomes of those early clinical trials for the treatment of solid tumors.

**Table 2:** CAR-T targets for treatment of solid tumors

Target	Condition	Sponsor	Clinicaltrials. gov identifier
CD133	CD133+ cancer	PLAGH	NCT02541370
CD70	CD70+ cancer	NCI	NCT02830724
CD171	Neuroblastoma	Seattle Children’s Hospital	NCT02311621
CEA	Liver metastases	Roger Williams Medical Center	NCT02850536 NCT02416466
	CEA+ cancer	Southwest Hospital, China	NCT02349724
cMet	Breast cancer	Upenn	NCT01837602
EGFR	EGFR+ solid tumors	PLAGH	NCT01869166
	Advanced glioma	RenJi Hospital	NCT02331693
EGFRvIII	GBM	Beijing Sanbo Brain Hospital	NCT02844062
	GBM	Duke University	NCT02664363
	EGFRvIII+ glioma	Upenn	NCT02209376
	Glioma	NCI	NCT01454596
EphA2	EphA2+ glioma	Fuda Cancer Hospital, Guangzhou	NCT02575261
EPCAM	Liver neoplasms Stomach neoplasms	Sinobioway Cell Therapy Co., Ltd. Sinobioway Cell Therapy Co., Ltd.	NCT02729493 NCT02725125
FAP	Malignant pleural mesothelioma	University of Zurich	NCT01722149
GD2	Neuroblastoma	Cancer Research UK	NCT02761915
	GD2+ solid tumors	NCI	NCT02107963
	Neuroblastoma	BCM	NCT01822652
	Neuroblastoma	Zhujiang Hospital	NCT02765243
	Neuroblastoma	BCM	NCT02439788
	Neuroblastoma	Children’s Mercy Hospital Kansas City	NCT01460901
	Sarcoma	BCM	NCT01953900
GPC3	GPC3+ HCC	Fuda Cancer Hospital, Guangzhou	NCT02723942
	HCC	Shanghai GeneChem Co., Ltd.	NCT02715362
	HCC	RenJi Hospital	NCT02395250
HER2	HER2+ cancer	Zhi Yang|Southwest Hospital, China	NCT02713984
	Breast cancer	Fuda Cancer Hospital, Guangzhou	NCT02547961
	HER2+ solid tumors	PLAGH	NCT01935843
	Sarcoma GBM HER2+ malignancies	BCM BCM BCM	NCT00902044 NCT01109095 NCT02442297 NCT00889954
	Head and neck cancer	King’s College London	NCT01818323
IL13Rα2	Glioma	City of Hope	NCT00730613 NCT01082926 NCT02208362
Mesothelin	Pancreatic cancer	Shanghai GeneChem Co., Ltd.	NCT02706782
	Metastatic PDAC, Epithelial ovarian Cancer, mesothelioma	Upenn	NCT02159716
	Metastatic PDAC	Upenn	NCT01897415 NCT02465983
	Breast cancer	MSKCC	NCT02792114
	Mesothelin+ cancer	PLAGH	NCT02580747
	Mesothelin+ cancer	NCI	NCT01583686
	Mesothelin+ cancer	MSKCC	NCT02414269
MUC1	MUC1+ cancer	PersonGen BioTherapeutics (Suzhou) Co., Ltd.	NCT02617134 NCT02587689 NCT02839954
MUC16	MUC16+ cancer	MSKCC	NCT02498912
MG7	Liver metastases	Xijing Hospital	NCT02862704
PSCA	Non-resectable pancreatic cancer	Bellicum Pharmaceuticals	NCT02744287
PSMA	Prostate cancer	MSKCC	NCT01140373
	Prostate cancer	Roger Williams Medical Center	NCT00664196
VEGFR2	Metastatic cancer, metastatic melanoma, Renal cancer	NCI	NCT01218867

Abbreviations: PLAGH, Chinese PLA General Hospital; NCI, National Cancer Institute; Upenn, University of Pennsylvania; BCM, Baylor College of Medicine; City of Hope, City of Hope National Medical Center; MSKCC, Memorial Sloan Kettering Cancer Center; GBM, glioblastoma multiforme; HCC, hepatocellular carcinoma; PDAC, pancreatic ductal adenocarcinoma

The ErbB family, subclass I of receptor tyrosine kinases (RTK), comprises four members widely expressed in adults at low levels: ErbB1/EGFR/HER1, ErbB2/HER2/Neu, ErbB3/HER3, and ErbB4/HER4 (Hynes and Lane 2005). Of these, EGFR and HER2 have been implicated in the development of a variety of tumors, including breast, lung, prostate, head and neck, pancreas, gastrointestinal tract, and gynecologic tract, so receptors have been intensely pursued as therapeutic targets (Whilding and Maher 2015). Several licensed monoclonal antibodies specific for EGFR (cetuximab, panitumumab, and nimotuzumab) and HER2 (trastuzumab and pertuzumab) are already available and demonstrate important therapeutic benefits. Moreover, these antibodies also present unique toxicities due to the baseline expression of EGFR or HER2 in normal tissues; for instance, most common are skin toxicity from EGFR inhibitors (Pastore et al., 2014) and cardiac toxicity associated with HER2-directed inhibitors, which is related to the physiological roles that EGFR and HER2 signaling is essential in the function of keratinocytes and cardiac myocytes. Taken together, there are grounds to believe that CAR targeting the ErbB family would have greater potential for potent antitumor activity in multiple malignancies; meanwhile, utmost consideration must be given to the safety concern as CAR-T cell-targeting EGFR/HER2 would possess greater avidity than the bivalent soluble antibody (Dotti et al., 2014).

Investigators at PLAGH pioneered an EGFR-directed CAR characterized by a shorted promoter in an effort to minimize the risk of on-target/off-tumor recognition and first tested this receptor in humans (NCT01869166). The preliminary outcome of 11 patients with advanced relapsed/refractory non-small cell lung cancer (NSCLC) who received anti-EGFR CAR-T cells at a dose of 0.45–1.09 × 10^7^ CAR^+^ cells/kg alone or following investigator’s choice conditioning chemotherapy showed that the treatment was well-tolerated without severe toxicity (Feng et al., 2016). As expected, mild skin toxicities due to on-target/off-tumor recognition were observed. In addition, objective responses including 2 PR lasting 2 to 3.5 months and 5 SD lasting 2 to 8(+) months were observed. Of note, immunohistochemistry (IHC) examination of biopsy tumor tissues from patients achieving either PR or SD illustrated that anti-EGFR CAR-T cells could traffic to tumor sites and infiltrate the tumor tissues and elicit EGFR-specific cytotoxicity even at 3.5 months post-cell infusion, implying that anti-EGFR CAR-T cells could persist and remain functional in an immunosuppression microenvironment. On this basis, this receptor in addition to a conditioning chemotherapy regimen of Cy and Nab-paclitaxel in order to eradicate stroma in other EGFR-positive solid tumors including cholangiocarcinoma (CCA) and pancreatic cancer (PC) are being tested.

Investigators at NCI first conducted a clinical trial to test anti-HER2 third-generation CAR with a CD28.CD137.ζ endodomain in patients with metastatic cancer (NCT00924287). Unfortunately, the first patient with metastatic HER2^+^ colon cancer who received 10^10^ T cells (79% CAR^+^) following conditioning chemotherapy developed rapid respiratory distress within 15 min after cell infusion and ultimately died of multiple organ failure as a result of reactivity against lung epithelial cell expression of low levels of HER2 (Morgan et al., 2010). This unforeseen systemic adverse event has been known as a fatal example of the on-target/off-tumor effect of CAR-T cells targeting non-tumor-specific antigens and lends a cautionary tale to using CAR-T cells in solid tumors. However, encouraging safety data from nine patients with osteosarcoma who received 28ζ CAR targeting HER2 transduced T cells at doses ranging from 10^4^–10^6^ cells/m^2^ without conditioning (NCT00902044) were reported at the American Association for Cancer Research (AACR) 2012 by Ahmed et al. at BCM (Ahmed et al., 2014). Infusion was well tolerated without systemic side effects and no elevation of pro-inflammatory cytokine. Updated results of this trial in the first 19 patients with HER2-positive sarcoma (including 16 osteosarcomas) further confirmed that anti-HER2 CAR-T cell treatment was safe and feasible as no dose-limiting toxicity (DLT) was observed even in the highest dose level of 1 × 10^8^ CAR^+^ T cells/m^2^ (Ahmed et al., 2015). Four of seventeen evaluable patients had SD for 12 weeks to 14 months, and 3 had their residual tumor removed with no further treatment and remained in remission at 6, 12, and 16 months, albeit no post-infusion expansion of anti-HER2 CAR-T cells in the PB was observed in most of the treated patients. Multiple reasons may contribute to the difference in the observed toxicity profile between the BCM and NCI trials, including no prior conditioning chemotherapy, 2-log lower maximum dose of cells, using a 28ζ CAR rather than CD28.4-1BB. ζ CAR. Furthermore, the HER2-specific scFvs of each CAR were derived from different MAbs (FRP5 vs. trastuzumab), which also could account for the substantial differences in safety observed in both trials. Besides sarcoma, Ahmed et al. also conducted a trial of HER2-specific CAR-T cells for GBM (NCT02442297). Furthermore, another two trials determining the safety of virus-specific HER2 re-targeted CAR-T cells (CMV; NCT01109095) (EBV; NCT00889954) are ongoing at BCM.

Variant III of the epidermal growth factor receptor (EGFRvIII), the most common variant of EGFR first identified in human GBM, is also present in many other tumor types, but is not found in healthy tissues (Li and Wong, 2008), which makes EGFRvIII a suitable target for CAR-T cell therapy. Investigators at Upenn reported (NCT02209376) (O’Rourke et al., 2016) the initial outcome of the first 9 patients with EGFRvIII-positive GBM who were treated with anti-EGFRvIII CAR-T cells at a dose of 1–5 × 10^8^ CAR^+^ cells. The infusion was safe without evidence of off-tumor toxicity or CRS and no cross-reactivity to wild type EGFR, except one patient developed non-convulsive status epilepticus 9 days after infusion, which was resolved with standard treatment and anti-cytokine therapy. Significant expansion of anti-EGFRvIII CAR-T cells between 7 and 10 days post-infusion were observed in all patients, which is a sharp contrast to what Ahmed et al. observed (Ahmed et al., 2015). More importantly, a pathologic evaluation of five patients who had undergone surgical resection of tumors between 6 and 120 days after infusion demonstrated that anti-EGFRvIII CAR-T cells were immunologically active as recruitment of new T cells as well as specific EGFRvIII target antigen loss in GBM cells were observed in some cases. EGFRvIII-specific CARs for patients with GBM are also being tested at several other institutions, including the NCI (NCT01454596), Beijing Sanbo Brain Hospital (NCT02844062), and Duke University (NCT02664363).

MSLN is a tumor-associated antigen named for its low-level expression on mesothelial cells that line the peritoneal, pleural, and pericardial cavities, and yet is overexpressed in malignant pleural mesothelioma, pancreatic, ovarian, and lung cancer (O’Hara et al., 2016). To minimize the potential on-target/off-tumor toxicity of MSLN-specific CAR, investigators at Upenn developed an approach to transiently express the CAR on T cells by using electroporation of CAR mRNA and tested the safety of multiple infusions of MSLN-RNA-CAR-T cells in a first in-human study (NCT01355965) based on the encouraging results of preclinical studies (Zhao et al., 2010; Barrett et al., 2011). Preliminary results in four patients (three malignant pleural mesothelioma, one pancreatic adenocarcinoma) showed that multiple infusions of MSLN-RNA-CAR-T cells was feasible and safe without overt evidence of on-target/off-tumor toxicity against normal tissues, except one patient developed anaphylaxis due to the murine scFv used in the CAR and went into cardiac arrest within minutes of completing the third infusion but rapidly recovered as a consequence of intensive treatment (Maus et al., 2013; Beatty et al., 2014). Researchers also demonstrated the antitumor activity of anti-MSLN CAR-T cells based on the clinical and laboratory evidence such as specific and potent lysis capacity of anti-MSLN CAR-T cells resulting in a decrease in the tumor cells in a patient’s ascites. Updates of another trial to evaluate anti-MSLN CAR-T cells in patients with pancreatic ductal adenocarcinoma (PDAC) (NCT01897415) were presented by Beatty et al. (2015). Well-tolerated toxicity and modest antitumor efficacy with 2 SD among 6 treated patients were demonstrated. In light of the aforementioned safety profile of anti-MSLN CAR-T cells, researchers opened a trial to test anti-MSLN CAR-T cells transduced with lentivirus for PDAC, epithelial ovarian cancer, and malignant epithelial pleural mesothelioma (NCT02159716), and reported the early results of this trial at AACR 2015 (Tanyi et al., 2015). Five patients with advanced stage cancers (two serous ovarian, two epithelial mesothelioma, and one PDAC) were treated with a single dose of 1–3 × 10^7^ CAR^+^ T cells/m^2^ without lymphodepletion. Infusions were well tolerated with no acute AE and no evidence of on-target/off-tumor toxicity albeit anti-MSLN CAR-T cells were found to traffic to on-target/off-tumor sites such as the pericardial fluid. The loss of malignant cells in the pleura fluid near 4 weeks post-cell infusion happened in one patient and another experienced stable to decreased burden of disease, suggesting anti-MSLN CAR-T cells possessed direct anti-tumor efficacy. Updated experiences of 6 patients with recurrent serous ovarian cancer were reported by Tanyi et al. at ASCO 2016 (Tanyi et al., 2016). The treatment still was well-tolerated even in two patients who received 3 × 10^8^ CAR^+^ T cells/m^2^. Six of six treated patients achieved SD 1 month after anti-MSLN CAR-T cell infusion, and clearance of pleural effusion by anti-MSLN CAR-T cells which trafficked to tumor sites was noted in one patient. Moreover, a variety of MSLN-specific CARs are being tested in other ongoing trials (NCT02414269, NCT01583686, NCT02465983, and NCT02792114).

Prostate-specific membrane antigen (PSMA), a type II transmembrane glycoprotein, is expressed in all forms of prostate tissue, but is upregulated 10-fold in prostate cancer (Ma et al., 2004). PSMA-targeted CAR-T cells for patients with castrate metastatic prostate cancer (CMPC) was tested in a dose escalation study performed at MSKCC (NCT01140373), and early experiences in the first three patients who received dose level 1 (1 × 10^7^ CAR^+^ T cells/kg) following 300 mg/m^2^ of Cy one day were presented at ASCO 2012 by Slovin et al. No toxicities occurred, and two of three treated patients had SD for longer than 6 months (Slovin et al., 2012). On this basis, the fourth patient received the same dose with a modified vector with a higher copy number, and an additional three patients in cohort 2 were treated with 3 × 10^7^ CAR^+^ T cells/kg following the same conditioning regimen. Updated results showed that one of two patients achieving SD in cohort 1 maintained a response for greater than 16 months. All three patients in cohort 2 had elevated levels of cytokine, including interleukin-4 (IL-4), interleukin-8 (IL-8), and IL-6, etc., and up to 2 weeks persistence of CAR-T cells post-CAR-T cell infusion (Slovin et al., 2013). An encouraging early data from trial of PSMA-specific CAR (NCT00664196) was demonstrated by Junghans (Junghans, 2012) at ASCO 2012 as two PR with a decrease in prostate specific antigen (PSA) levels and delayed disease progression were attained among five patients with metastatic prostate cancer who were treated with a non-myeloablative (NMA) conditioning regimen followed by either 10^9^ or 10^10^ anti-PSMA CAR-T cells and IL-2 given by continuous infusion for 1 month alongside the T cell infusion. Of note, these two PRs were observed at the lowest T cell dose of 10^9^, together with the plasma IL-2 in non-responders was as much as 10-fold lower compared to that in responders, the researchers drew a conclusion that adequate higher IL-2 *in vivo* plus a higher CAR-T cell dose could be beneficial to anti-tumor activity of anti-PSMA CAR-T cells, which is being tested in a redesign study.

CEA is overexpressed in many epithelial cancers but is also expressed in a variety of normal epithelial cells (Hammarstrom, 1999). Investigators at Roger Williams Medical Center conducted a phase I hepatic immunotherapy for metastases (HITM) trial (NCT01373047) to investigate CAR-T cell targeting CEA for patients with CEA-expressing adenocarcinoma liver metastases and reported the early results in 2015 (Katz et al., 2015). Given that severe transient colitis had been induced by intravenous infusion of CEA-specific TCR-transduced T cells in a previous study (Parkhurst et al., 2011), a regional delivery strategy was adopted aiming to enhance the tolerability and therapeutic efficacy of anti-CEA CAR-T cells. Of the six treated patients, three received anti-CEA CAR-T cells alone in dose-escalation fashion (10^8^, 10^9^, and 10^10^ cells), whereas an additional three patients received the maximum planned anti-CEA CAR-T cell dose (10^10^ cells × 3) along with systemic IL-2 support. No grade 3 or 4 AE related to the anti-CEA CAR-T cell infusion developed in all treated patients. One patient had stable disease for 23 months after anti-CEA CAR-T cell infusion and other five patients had progressive disease; however, a median 37% decrease of CEA levels was observed in patients receiving systemic IL-2 support, and four of six treated patients showed necrosis of metastatic liver lesions. Another anti-CEA CAR-T cell for CEA positive cancer is currently being tested at Southwest Hospital in China (NCT02349724); no results of this trial have been published yet.

IL13Rα2, a monomeric high-affinity IL13 receptor, is selectively expressed on GBM while absent in the surrounding normal brain tissue, rendering it can be proposed as an optimal candidate for target selection of CAR-T cell therapy in glioma (Thaci et al., 2014). Building on their previous experience in 3 patients with glioblastoma that multiple intracranial infusions of first generation CAR-T cells targeting IL13Rα2 was well-tolerated (Brown et al., 2015), the investigators at City of Hope National Medical Center (City of Hope; USA) conducted a trial (NCT02208362) to evaluate a IL13Rα2-specific BBζ CAR (IL13 BBζ-CAR) without lymphodepleting chemotherapy in GBM and reported their clinical experience in one patient with recurrent multifocal GBM (Brown et al., 2016). Local control after intracavitary administration of six cycles of IL13BBζ-CAR T cells was observed, whereas other disease foci that were distant from the CAR-T cell injection site continued to progress. Together with new metastatic lesions in the spine, ten additional intraventricular treatment cycles were administered in an effort to effectively control tumor progression at distant sites. After five intraventricular infusions, all tumors including spinal metastases have decreased by 77%–100% and continued to resolve during the five additional intraventricular consolidation infusions. No grade ≥3 toxicities related to intracranial infusions of IL13 BBζ-CAR-T cells occurred during these infusions. The clinical response lasted for 7.5 months, however, the disease eventually recurred at new four lesions. The researchers speculated that this relapse might be attributed to the downregulation of IL13Rα2, which should be an example of tumor editing caused by the selective pressure exerted by CAR-T cell therapy.

## TOXICITIES OF CAR-T CELL THERAPY

CAR-T cells offer a promising new therapy for cancers, but the toxicities elicited in the clinical trials are still a great concern. Deaths with CAR-T were reported previously (Morgan et al., 2010; Brentjens et al., 2010) and recently (DeFrancesco 2016), which has been a wake-up call to the potential for toxicity of CAR-T cell therapy (Junghans 2010). The toxicities of CAR-T cell therapy generally fall into several of the following categories.

### Cytokine release syndrome

The most significant and life-threatening toxicity following CAR-T cell therapy is CRS, which is attributable to the rapid and extensive activation of infused CAR-T cells upon antigen engagement and results in elevated inflammatory cytokines (Lee et al., 2014). The frequency and severity of CRS vary greatly among different studies, which has been reported in 18%–100% of patients, with sCRS noted in 27%–53% of patients (Batlevi et al., 2016). Since CRS can be successfully ameliorated with the IL-6R inhibitor tocilizumab (Grupp et al., 2013), investigators now have more experience in how to diagnose and manage CRS, and recently several reviews have highlighted and summarized these advances (Lee et al., 2014; Maude et al., 2014b; Xu and Tang 2014; Brudno and Kochenderfer 2016; Bonifant et al., 2016). The strong correlation between the severity of CRS and the tumor burden at the time of infusion has been well-recognized (Maude et al., 2015a), nevertheless, whether to use prophylactic or early tocilizumab remains undetermined (Nellan and Lee 2015). Besides tocilizumab, other cytokine-directed approaches to managing CRS could be considered, and inhibitor of TNF-α infliximab has also been successfully used in our center. Of note, due to the concern that the pre-emptive CRS treatment could impair the anti-tumor efficacy of the infused CAR-T cells, Ruella et al. added kinase inhibitor ibrutinib to anti-CD19 CAR-T cells in an effort to prevent CRS, and proved the feasibility of this strategy in an NOD/SCID/gamma-chain-deficient (NSG) mice model. On this basis, a clinical trial (NCT02640209) was opened to test CTL019 cells in addition to ibrutinib in patients with CLL (Ruella et al., 2017). Significantly, a more complete understanding regarding the biology of the syndrome and to subsequently prevent or abrogate sCRS as well as to determine predictive biomarkers for CRS is of utmost importance. David et al. developed a novel algorithm to predict CRS recently and showed that peak levels of IFN-γ, IL-6, sgp130, and sIL-6R within the first month after infusion could be proposed as a predictive marker for sCRS, which might guide future cytokine-directed therapy (Teachey et al., 2016).

### On-target/off-tumor toxicity

This type of toxicity is a direct result of the specific recognition of a target expressed in normal tissues by CAR-T cells, thus its profile is dependent on the antigenic specificity of the engineered T cell and can be predictably seen in a variety of organ systems (Bonifant et al., 2016; Barrett et al., 2015). B cell aplasia is a classical on-target/off-tumor toxicity in patients treated with anti-CD19 or 20 CAR-T cells (Maude et al., 2014a; Zhang et al., 2016a; Kochenderfer et al., 2012), which can easily be managed with intravenously (i.v.) Ig replacement and serve as a surrogate maker for the persistence of CAR-T cells *in vivo* (Maude et al., 2014a). With respect to solid tumors, the toxicity resulting from on-target/off-tumor recognition may not be so tolerable and acceptable, which was highlighted by an aforementioned anti-HER2 CAR-T death case report (Morgan et al., 2010). However, subsequent trials of ErbB family-specific CAR demonstrated acceptable toxicities (Ahmed et al., 2015; Feng et al., 2016). In order to minimize the risk of on-target/off-tumor toxicity in solid tumors, multiple strategies have been developed and fall in to two major categories: enhancing selectivity of CAR aiming to enhance the tumor recognition and bystander discrimination as well as control CAR-T cell activity in an attempt to provide ways for physician to intervene and either eliminate or modulate the T cell activity when acute severe off-tumor toxicities occurred. The enhancing selectivity of CAR can be achieved via selecting safer antigen (i.e., tumor specific antigen EGFR vIII, aberrantly glycosylated antigens, TCR-like CAR) (O’Rourke et al., 2016; Posey et al., 2016; Zhang et al., 2014; Ma et al., 2016a; Liu et al., 2017a), combinatorial antigen targeting (i.e., complementary signaling, synNotch/CAR circulation, iCAR) (Fedorov et al., 2013; Kloss et al., 2013; Roybal et al., 2016; Wilkie et al., 2012), turning sensitivity of scFv by turning the affinity (Caruso et al., 2015; Liu et al., 2015), and masked CAR (Desnoyers et al., 2013), while the design of limiting CAR expression (i.e., transient mRNA CAR) (Maus et al., 2013; Beatty et al., 2014), switchable CAR-T cell (i.e., dimerizing small molecules, tumor targeting antibody) (Cao et al., 2016; Juillerat et al., 2016; Ma et al., 2016b; Rodgers et al., 2016; Wu et al., 2015) and suicide gene (i.e., inducible Caspase-9, antibody-mediated depletion) (Turtle et al., 2016a, b; Di Stasi et al., 2011) can be introduced to flexibly control the CAR-T cell activity. For a detailed description and analysis of these proof-of concept designs, please refer to the reviews published recently by our group (Wang et al., 2017b) and Upenn group (Lim and June 2017). Overall, most of these strategies are in early stages, the preliminary results from the experimental studies provide the initial evidence of feasibility and pave the road to further optimization. However, the eventual effects of these novel designs still need to be determined in forthcoming clinical trials.

### Neurologic toxicities

Neurologic toxicities were described in 13%–52% of patients across institutions, and symptoms ranged from confusion and delirium to aphasia, obtundation, myoclonus, and seizure, which is frequently self-limiting (Maude et al., 2014a; Davila et al., 2014; Lee et al., 2015a; Kochenderfer et al., 2015; Brentjens et al., 2013; Park et al., 2015; Grupp et al., 2015; Schuster et al., 2015). However, the deaths of 3 patients with R/R B-ALL after receiving anti-CD19 CAR-T cells following Cy/Flu conditioning chemotherapy highlights the potential harm of this type of toxicity (DeFrancesco, 2016). The etiology of this syndrome remains unclear; it often accompanies CRS, but also can be present alone (Maude et al., 2014a). The NCI believes that tocilizumab may temporarily worsen neurotoxicity, thus they recommend using high-dose steroids rather than tocilizumab to treat grade ≥3 neurologic toxicity (Brudno and Kochenderfer, 2016). More studies are needed to determine the pathophysiology and subsequently find the best approach to treat or prevent severe neurotoxicity.

### Other rare toxicities

Hemophagocytic lymphohistiocytosis/macrophage activation syndrome (HLH/MAS) occurred in a subset of patients who received CAR-T cells (Porter et al., 2015; Grupp et al., 2013). HLH/MAS is a rare AE triggered by a cascade of immune activation and characterized by hyperinflammation with prolonged fever, hepatosplenomegaly, and cytopenias. This syndrome has parallels in both clinical and laboratory findings with CRS; however, elevated levels of ferritin and triglycerides can be used to differentiate these two syndromes (Janka, 2012). Genetic predisposition may increase the risk of developing HLH/MAS in some patients (Maude et al., 2014b), but it can be well controlled by tocilizumab.

An IgE-mediated clinical anaphylaxis after the third MSLN-RNA CAR-T cell infusion has been reported, which was suggested by markedly elevated tryptase levels and the presence of human anti-mouse antibodies after cell infusion (Maus et al., 2013). This effect may be attributable to the multiple infusion schedule, which may lead to a substantial humoral immune response against the CAR with murine SS1 scFv. Similarly, a cellular immune response specific for murine scFv epitopes of anti-CD19 CAR was identified in the patients who received the second infusion, resulting in the failure of the second infusion (Turtle et al., 2016a, b). We also observed that anti-EGFR CAR-T cells could not be well proliferated in some patients who received a second infusion. Humanized or fully human scFv will hopefully abrogate or at least reduce the potential for anti-murine immune-mediated rejection, which has been shown to be highly effective in a phase I study of humanized CD19-directed CAR-T cells (CTL119) (Maude et al., 2015b).

Tumor lysis syndrome (TLS) also has been described in some patients (Dai et al., 2015; Grupp et al., 2013; Brudno et al., 2016). A severe TLS occurred after stand-alone low-dose chemotherapy in a patient who relapsed after anti-CD19 CAR-T cell infusion resulting from the loss of CAR-T cells (Zhang et al., 2016b).

## CHALLENGES FOR CAR-T CELL THERAPY

CAR-T cell therapy has shown unprecedented initial response rates in advanced B cell malignancies; however, relapse after CAR-T cell infusion is a major hurdle in successful CAR regimens. To date, the main understanding regarding this phenomenon is gained from the trials involving CD19, and two modes of relapse have been seen: CD19 negative and CD19 positive (Maude et al., 2015a). CD19 negative relapses were reported by several groups (Maude et al., 2014a; Lee et al., 2015a; Turtle et al., 2016a; Grupp et al., 2013; Pegram et al., 2015), in which the Upenn group showed the highest incidence of up to 60% (Ruella et al., 2016). This group also demonstrated that splice-based adaptations in tumor cells was an underlying mechanism for tumor antigen loss escape, leading to an outgrowth of tumor escape variant cells (Sotillo et al., 2015). Dual-targeted T cells is a potential strategy to reduce the risk of antigen escape, which has two patterns: (i) T cell expressing a CAR comprising two different scFv in tandem (termed “TanCAR”) (Grada et al., 2013) or expressing two different CARs targeting two different targets (known as “dual-signaling CAR”) (Ruella et al., 2016). Both of these two designs only have one group CAR-T cells; (ii) “pooled” CAR-T, where two groups of CAR-T cells express two different CARs, which can be infused sequentially or simultaneously. Although the preliminary evidence of feasibility of TanCAR and dual-signaling CAR designs were demonstrated in several proof of concept preclinical studies (Zah et al., 2016; Grada et al., 2013; Hegde et al., 2016), which are challenging to implement due to the difficulty of identifying 2 appropriate targets on 1 tumor (Jackson and Brentjens 2015) as well as the constraint of suitable epitopes selection in the setting of TanCAR (Sadelain, 2016). Regarding the “pooled” CAR-T, the development period is longer as is the combination of two groups of CAR-T cells. CD19 positive relapse as a result of loss of CAR-T cell persistence can be prevented by prolonging CAR-T cell persistence, which can be achieved by using preconditioning, optimization of CAR constructs, and increasing the ratio of early lineage T cells (Maude et al., 2015a). Multiple infusions of CAR-T cells is also an effective option for patients who experienced CD19 positive relapse (Maude et al., 2014a; Kochenderfer et al., 2012, 2015). However, failure of the second or third infusion was observed in a subset of patients (Lee et al., 2015a; Turtle et al., 2016a, b), warranting further studies.

It remains a huge challenge for CAR-T cell therapy beyond the hematological malignancies. Besides the aforementioned safety concern due to the risk of on-target/off-tumor recognition, limited therapeutic success is another major hurdle in CAR-T cell treatment of solid tumors. This limitation is mainly attributable to the hostile solid tumor microenvironment characterized by physical/anatomical barriers (i.e., tumor stroma) and immunosuppressive cytokines and immune cells such as regulatory T (T_REG_) cells and myeloid-derived suppressor cells (MDSCs), which are harmful to the infiltration of infused CAR-T cells into tumor sites and for retaining cytotoxic functionality (Newick et al., 2016). One promising approach to circumvent this obstacle is the use of armored CAR-T cells, which are the fourth-generation CAR-T cells that are further modified to additionally express immune-modulatory proteins, including cytokines (IL-2, IL-12, and IL-15) and ligands (PD-1/CD28 fusion, CD40L, or 4-1BBL) (Fesnak et al., 2016; Khalil et al., 2016). Armored CAR-T cells modified to secrete pro-inflammatory IL-12 have been known as TRUCKs (T cells redirected for universal cytokine killing), which can release IL-12 upon CAR-mediated T cell activation and have yielded encouraging results in several preclinical studies (Chmielewski et al., 2014). The first clinical trial to explore the impact of IL-12 CAR-T cells has been opened by MSKCC (NCT02498912), where IL-12-secreting CAR-T cells transduced with a 28ζ CAR targeting mucin-16 (MUC-16) is being tested in patients with ovarian cancer. Other examples of armored CAR-T cells such as those modified to additionally express ligands are in the proof-of-concept stage and have not yet moved forward to the stage of clinical trials (Khalil et al., 2016).

CAR-T cell therapy is entering advanced phases of clinical trial testing; anti-CD19 CAR-T especially will enter mainstream clinical oncology for patients with B cell malignancies in the near future (Klebanoff et al., 2016). However, these clinical successes thus far have employed autologous cells, which were produced on the campuses of multiple academic facilities for a given recipient on a case-by-case basis. This personalized manufacturing and widely “distributed” approach greatly limit the broad implementation and commercialization of CAR-T cell therapy due to the complicated and time-consuming procedures, great cost to generate one product for one patient, and heterogeneity of T cell products produced for or from individual recipients (Torikai and Cooper 2016). Centralized manufacturing of patient-derived CAR-T cells and distribution to multiple points-of-care have already been adopted by biopharmaceutical companies such as Novartis, Juno, Kite, and CBMG, aiming to reduce the variation of CAR-T cell products and the costs associated with them (Cooper 2015). On this basis, “off-the-shelf” (OTS) CAR-T cell therapy, which is deemed to be the ultimate product formulation, will open of a new chapter in the race to commercialize CAR-T cell therapy (Ratner 2016). OTS CAR-T cell (also known as universal CAR-T cell, or UCAR-T) is defined as a biologic that is pre-prepared in advance from one or more healthy unrelated donors, validated, and cryopreserved (Torikai and Cooper 2016) and then can be shipped in a day or two to patients worldwide. The first clinical application of universal CAR-T cells was reported by Qasim et al. (2015) at ASH 2015; a 1-year-old girl with relapsed leukemia achieved molecular remission without significant toxicity after transcription activator-like effector nucleases (Talen) engineered anti-CD19 UCAR-T cell infusion following lymphodepleting conditioning with Flu 90 mg/m^2^, Cy 1.5 g/m^2^, and alemtuzumab 1 mg/kg, providing early proof-of-concept evidence for this strategy. However, more studies are needed to optimize this innovative approach, as many challenges remain (Torikai and Cooper 2016).

## CONCLUSIONS AND FUTURE PERSPECTIVES

CAR-T cell therapy, especially CD19-specific CAR-T cell therapy, is poised to shift the treatment paradigm for B cell malignancies as significant response rates and well-tolerated toxicities. For this reason, many researchers are currently developing strategies in an effort to recapitulate this success in solid tumors, albeit the road is unlikely to be straightforward mainly due to the risk of on-target/off-tumor recognition and hostile solid tumor microenvironments resulting in less efficacy. However, strategies are being implemented to address these obstacles, and some encouraging preliminary results have been demonstrated. Despite these advancements, several issues remain to be resolved (Box 1), providing impetus for continuous optimization of CAR as well as appropriately powered, well-designed clinical studies.

**Table:** 

Box 1: Unresolved questions in CAR-T cell therapy
· What is the suitable dosage range of CAR-T cells, and is it the same in different targets or diseases?
· What is the optimal ratio of engineered CAR-T cell subsets, including early memory T cells?
· How great immunogenicity of CAR-modified T cells can be resolved by humanized and or fully human CAR, and what is the optimal multiple infusion regimen?
· Can smart CAR aiming to reduce on-target/off-tumor recognition provide for adequate safety in clinical testing?
· What is the optimal management for patients who have received CAR-T cell therapy, and what are the relative roles of CAR-T cells and HSCT in the context of transplant-eligible patients?

Actually, besides how to enhance efficacy and safety of CAR-T cells, the development of resistance is particularly noteworthy for the optimization of CAR-T cell therapy either in hematological malignancies or solid tumors. As is well known, downregulation of target antigens is one of mechanism that tumor escape from cancer immunotherapy (Marincola et al., 2000; Leen et al., 2007). By tumor editing such as target antigen loss (Evans et al., 2015), mutation (Sotillo et al., 2015) or leukemic lineage switch (Gardner et al., 2016), tumor clone can be invisible to the CAR-T cell therapy, resulting in the tumor cells resistant to the killing mediated by CAR-T cells and disease recurrence. This phenomena occurs not only in hematological malignancies but also in solid tumors (Brown et al., 2016; Hegde et al., 2016), highlighting the shortcoming of single-target CAR-T cell therapy. Generating T cells capable of recognizing multiple antigens may be an effective alternative to address the challenge of resistance and relapse after CAR-T cell therapy; moreover, T cell exhaustion, an acquired state of T cell dysfunction due to the persisting antigenic stimulation during cancer, can also lead to the CAR-T cells failure to eliminate the tumor cells even the target antigens are still present. PD-L1/PD-1 immune inhibitory axis plays a central role in the regulation of T cell exhaustion. Blocking PD-1 can re-invigorate the exhausted T cells and improve control of cancer, which has been seen in a patient with refractory DLBCL whose disease has progressed after anti-CD19 CAR-T cell infusion (Chong et al., 2017). Thus we believe that elucidating the underlying mechanisms of CAR-T cell therapy resistance and development of effective combination therapy that combination of PD-1/PD-L1 blockade with CAR T cell therapy in an effort to reverse T cell exhaustion will be an active research area in CAR-T cell therapy field.

Finally, with the emergence of clustered regularly interspaced short palindromic repeats (CRISPR) and CRISPR-associated protein 9 (Cas9) system, a new gene editing tool that can induce targeted genetic alterations and process multiplex genome engineering with a relative ease compared to the Talen system (Maeder and Gersbach 2016; Cong et al., 2013), applying this novel system to disrupt TCRα subunit constant (TRAC) and or beta-2 microglobulin (B2M) of CAR-T cells to avoid GVHD and minimize immunogenicity has been actively investigated (Liu et al., 2017b; Eyquem et al., 2017; Ren et al., 2016). The preliminary data from these experimental studies suggest that CRISPR-Cas9-mediated multiplex gene editing is readily applicable to CAR-T cells even in the setting of triple genes disruption. This would be helpful for the development of OTS donor-derived CAR-T cells. We believe the combination of CAR-T cell therapy and gene editing will revolutionize the industry even if many difficult challenges lie ahead.
